# The critical role of DNA damage‐inducible transcript 4 (DDIT4) in stemness character of leukemia cells and leukemia initiation

**DOI:** 10.1002/1878-0261.70090

**Published:** 2025-07-07

**Authors:** Yishuang Li, Zhijie Cao, Haiyan Xing, Zhenya Xue, Wenbing Liu, Jiayuan Chen, Yihan Mei, Runxia Gu, Hui Wei, Shaowei Qiu, Min Wang, Qing Rao, Jianxiang Wang

**Affiliations:** ^1^ State Key Laboratory of Experimental Hematology, National Clinical Research Center for Blood Diseases, Haihe Laboratory of Cell Ecosystem, Tianjin Key Laboratory of Cell Therapy for Blood Diseases, Institute of Hematology & Blood Diseases Hospital Chinese Academy of Medical Sciences & Peking Union Medical College Tianjin China; ^2^ Tianjin Institutes of Health Science China

**Keywords:** acute myeloid leukemia, DDIT4, HOXA, leukemogenesis, self‐renewal, stemness

## Abstract

Leukemia stem cells (LSCs) are critical for leukemia initiation, and the stemness properties of LSCs are related to disease relapse. Stemness properties, including quiescence, self‐renewal, and chemoresistance, are maintained through an interplay between leukemia cells and the bone marrow (BM) niche. Here, we demonstrated that DNA damage‐inducible transcript 4 (DDIT4) can be induced in a hypoxic BM niche and is required for the quiescence and self‐renewal of AML1‐ETO9a (AE9a)‐transformed leukemia cells *in vitro*. More importantly, analysis of publicly available transcriptional data from adult acute myeloid leukemia (AML) patients revealed that elevated DDIT4 expression correlates with poor prognosis. Furthermore, DDIT4 knockout markedly suppressed leukemia initiation, quiescence, chemoresistance, and self‐renewal of AE9a‐transformed leukemia cells *in vivo*. Mechanistically, DDIT4 upregulates the expression of HOXA gene cluster, and re‐expression of *HOXA6* in DDIT4 knockout AE9a cells can rescue the impaired leukemia initiation. Our findings demonstrate the critical role of DDIT4 in the stemness of AE9a leukemia cells and elucidate its underlying mechanism, suggesting that targeting DDIT4 may represent a promising therapeutic strategy for eliminating LSCs in AML1‐ETO leukemia.

## Graphical abstract

Stemness properties, including quiescence, self‐renewal, and chemoresistance, are closely associated with leukemia relapse. Here, we demonstrate that DNA damage‐inducible transcript 4 (DDIT4) is induced in the hypoxic bone marrow niche and is essential for maintaining the stemness of AML1‐ETO9a leukemia cells. DDIT4 plays a critical role in leukemia initiation. Mechanistically, the HOXA gene cluster mediates DDIT4's function in leukemia.

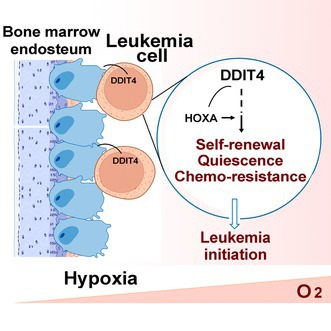

**Abbreviations**
AE9a: AML1‐ETO9a
AML: acute myeloid leukemia
BM: bone marrow
CBM: central bone marrow
DDIT4: DNA damage‐inducible transcript 4
DNR: daunorubicin
EBM: endosteal surface of bone marrow
ELDA: extreme limiting dilution analysis
FACS: fluorescence‐activated cell sorting
HSPC: hematopoietic stem and progenitor cell
LSCs: leukemia stem cells
MSC: multipotent stromal cells
OBC: mouse calvarial osteoblast

## 1 Introduction

Accumulating evidence indicates that acute myeloid leukemia (AML) is initiated and maintained by a rare subpopulation that was identified as leukemia stem cells. Although the stemness properties of leukemia cells, including self‐renewal, cell cycle quiescence, and chemoresistance [1, 2] have been established, the molecules and pathways driving leukemia cells stemness remain to be fully addressed. Self‐renewal potential is critical for leukemia initiation, as well as its recurrence after initial treatment, which eventually leads to disease relapse [3, 4]. LSCs exhibit slow cycling and are maintained in G0 phase; this quiescence property endows leukemia cells with resistance to chemotherapy [5, 6]. It is well accepted that the stemness properties of leukemia cells are associated with the poor survival of AML patients [7, 8, 9].

Leukemia was previously thought to be driven by intrinsic genetic or epigenetic alterations in hematopoietic cells. However, it has been recognized that changes in the bone marrow (BM) niche can promote leukemogenesis and chemoresistance [10, 11]. It was reported that AML cells engrafted in the BM endosteal area exhibited chemotherapy resistance after transplantation of human AML LSCs using the xenograft model [12]. Several studies also suggested that the quiescence and chemoresistance were maintained by interaction between leukemia cells and the BM niche, which was mediated by some adhesion molecules [13, 14].

Similar to normal hematopoietic stem cells (HSCs), LSCs reside at the hypoxic niche [15, 16]. The hypoxic environment is utilized to maintain the important functions of HSC, and appears to be required for LSC stemness [17]. The hypoxic microenvironment also mediates tumor progression and chemoresistance [18, 19]. To understand the molecular mechanisms responsible for maintaining the stemness of leukemia cells located in the hypoxic niche, in the present study, based on the AML1‐ETO9a (AE9a) leukemia mice model, we compared the gene expression profiles of leukemia cells in central BM with those in the counterparts isolated from the endosteal BM. As expected, upregulated genes in AE9a leukemia cells in the endosteal BM were enriched in the genes upregulated in response to hypoxia. Among these genes, the DNA damage‐inducible transcript 4 (*Ddit4*) gene was significantly overexpressed in AE9a leukemia cells in the endosteal BM.

DDIT4 can be induced by hypoxia and energy stress. As a negative regulator of mTOR, DDIT4 plays an essential role in triggering autophagy under cellular stress [20]. Although it has been reported that high DDIT4 expression is related to a poor prognosis in several types of solid tumors and leukemia [21, 22, 23], the mechanism and its role in leukemogenesis and leukemia cell stemness have not been elucidated yet. Herein, we investigated the potential role and mechanism of DDIT4, which is upregulated in leukemia cells in endosteal BM, in leukemogenesis and maintaining quiescence and self‐renewal of leukemia cells. Our data provide evidence that DDIT4 plays a critical role in leukemia cell stemness maintenance and leukemia initiation.

## 2 Materials and methods

### 2.1 Mice and cell lines

All animal procedures were approved by the Institutional Animal Care and Use Committees of the State Key Laboratory of Experimental Hematology (License number: HCAMS‐DWLL‐NSFC2023115‐1). All experimental mice, including AE9a leukemia mice, wild‐type (*Ddit4* ^+/+^), and *Ddit4* ^−/−^ mice, were all in the C57BL/6 background and kept in a specific pathogen‐free unit with standard laboratory chow and drinking water. Six‐ to eight‐week‐old female C57BL/6 mice were purchased from Beijing Vital River Laboratory Animal Technology Co., Ltd (Beijing, China). Six‐ to eight‐week‐old *Ddit4* deficient (*Ddit4* ^−/−^) mice were purchased from Shanghai Model Organisms Center, Inc. (Shanghai, China).

All cell lines used in this study were purchased from the American Type Culture Collection (ATCC). All experiments were performed with mycoplasma‐free cells.

### 2.2 AML1‐ETO9a induced mouse AML transplantable model

AE9a induced mouse AML transplantable model was generated in our laboratory previously [24]. Briefly, c‐Kit^+^ HSPCs from mouse BM were infected by AE9a retrovirus and 2 × 10^5^ GFP^+^ cells were injected into the tail vein of lethally irradiated (9Gy) female C57BL/6 mice. The recipient mice developed leukemia on the 220th day after transplantation, and the AE9a induced mouse AML transplantable model was established. The mice displayed a lineage‐negative, c‐Kit‐positive phenotype with absent myeloid and lymphoid differentiation markers, accompanied by splenomegaly, confirming leukemia development. AE9a leukemia cells used in RNA sequencing analysis, DDIT4 expression assay, and analysis of chemoresistance were all from the third generation of transplanted mice, which developed leukemia with an average latency of 25 days after transplantation [25].

### 2.3 Preparation of endosteal bone marrow leukemia cells

Central and endosteal bone marrow cells were isolated as described [26, 27]. Bone marrow cells were flushed from the femurs and tibias of AE9a mice, which represent central bone marrow (CBM) cells. Femurs and tibias were then treated with 2 mg·mL^−1^ collagenase I (Sigma‐Aldrich, Darmstadt, Germany) at 37 °C for 15 min, and the cells were collected from the supernatant of digested bone fragments, which represent endosteal bone marrow (EBM) cells.

### 2.4 Isolation of primary mouse osteoblasts and multipotent stromal cells

Mouse calvarial osteoblasts (OBCs) and multipotent stromal cells (MSCs) were obtained according to a previously published procedure [24, 28]. Briefly, for OBCs, after the calvaria of 48‐h‐old neonatal mice were treated with collagenase for 20 min, OBCs were collected from the suspension and then maintained in α‐MEM medium supplemented with 15% FBS and 50 μg·mL^−1^ vitamin C. For MSCs, mouse BM cells were collected and cultured with DMEM/F12 medium with 15% FBS for 2 days. After removing nonadherent cells, the adherent cells were identified as positive for MSC‐specific markers and were used in a co‐culture assay.

### 2.5 RNA sequencing analysis

Isolated AE9a leukemia cells were processed for RNA extraction. The sequencing libraries were constructed using the rRNA‐depleted RNA by NEBNext^®^ Ultra™ Directional RNA Library Prep Kit for Illumina^®^ (NEB, Ipswich, MA, USA). After the evaluation of RNA‐seq libraries quality and cluster generation, the libraries were sequenced on an Illumina NovaSeq 6000 platform, and 150‐bp paired‐end reads were generated. The raw sequence data have been deposited in the GEO database under project accession number: GSE272010 and GSE272011.

### 2.6 Drug treatment for enrichment assay

1 × 10^6^ AE9a GFP^+^ cells were transplanted into sublethally irradiated C57BL/6 mice. On day 10 after transplantation, there were 10–20% GFP^+^ cells in peripheral blood. The mice in the Ara‐C treatment group received intraperitoneal injection of 500 mg·kg^−1^ Ara‐C once every 24 h for 3 days, and the mice in the control group were injected with PBS. The central and endosteal bone marrow cells were then collected 1 day after Ara‐C treatment, and GFP^+^ AE9a leukemia cells were sorted for further Ddit4 expression assay.

### 2.7 Generation of AE9a/*Ddit4* ^+/+^ and AE9a/*Ddit4* ^−/−^ cells

Bone marrow cells were obtained from 6‐ to 8‐week‐old *Ddit4* ^+/+^ (wild‐type, WT) or *Ddit4* ^−/−^ (*Ddit4* knockout) mice. c‐Kit positive cells were isolated using CD117 MicroBead Kit (Miltenyi Biotec, Bergisch Gladbach, Germany) and then transfected with AE9a retrovirus. After 48 h of culture posttransfection, GFP^+^ cells were sorted and used for subsequent co‐culture. GFP^+^ cells sorted from the cells transiently transfected with AE9a were designated as AE9a/*Ddit*4^+/+^ and AE9a/*Ddit4* ^−/−^. For leukemia development assay, 5 × 10^5^ GFP^+^ cells were injected into the tail vein of lethally irradiated (9Gy) C57BL/6 mice. Leukemia cells derived from transplanted mice that developed leukemia were designated as AE9a‐*Ddit*4^+/+^ and AE9a‐*Ddit4* ^−/−^.

### 2.8 Co‐culture of AE9a/*Ddit4* ^+/+^ and AE9a/*Ddit4* ^−/−^ cells with MC3T3‐E1 osteoblast cells

FACS sorted AE9a/*Ddit4* ^+/+^ and AE9a/*Ddit4* ^−/−^ cells were suspended in StemSpan SFEM (StemCell Technologies, Vancouver, BC, Canada) containing 20 ng·mL^−1^ mIL‐3, 20 ng·mL^−1^ mIL‐6, and 50 ng·mL^−1^ mSCF. MC3T3‐E1 osteoblast cells (2 × 10^5^ per well) were seeded in a 6‐well plate and cultured overnight in DMEM medium with 10% FBS. Subsequently, cell suspensions containing 1 × 10^6^ AE9a/*Ddit4* ^+/+^ or AE9a/*Ddit4* ^−/−^ cells were added onto MC3T3‐E1 cells in each well, respectively, and co‐cultured for 24 h (medium consists of SFEM and DMEM at volume ratio of 1 : 1), and then were used for G0 phase and colony formation assays.

### 2.9 G0 phase of cell cycle assay

Proportion of cells in G0 phase was determined as described previously [24]. Briefly, after the cells were stained with APC‐Ki67 and DAPI for 30 min, the proportion of cells in G0 phase was detected and analyzed by flow cytometry.

### 2.10 Colony formation assay

AE9a expressing cells were cultured in MethoCult M3434 (STEMCELL, Vancouver, BC, Canada) in 24‐well plates in triplicates (2 × 10^3^ per well) for 10 days. Counting of colonies, secondary, and tertiary colony‐forming unit cultures were performed as previously described [24].

### 2.11 Quantitative RT‐PCR

Total RNA was extracted using RNeasy Micro Plus kit (Qiagen, Hilden, Germany) and then reverse transcribed into cDNA. Quantitative RT‐PCR was performed on 7500 RT‐PCR System (Applied Biosystems, Thermo Fisher Scientific, Carlsbad, CA, USA) using SYBR Green PCR Master Mix. Expression levels of the target genes were normalized against GAPDH. The primer sequences are listed in Table S1.

### 2.12 Western blot analysis

10^6^ mouse AE9a leukemia cells were lysed, and equivalent amounts of protein were run on 10% SDS/PAGE and transferred to nitrocellulose membrane. Membranes were blotted using anti‐DDIT4 antibody (Proteintech, Wuhan, China).

### 2.13 Limiting dilution transplantation assay

AE9a‐*Ddit4* ^+/+^ and AE9a‐*Ddit4* ^−/−^ GFP^+^ leukemia cells were transplanted into sublethally irradiated recipient mice in one of the following doses: 10^5^, 10^4^, 10^3^, and 10^2^ cells. The leukemia initiating frequency was calculated using the extreme limiting dilution analysis (ELDA) method. All recipient mice were 6‐ to 8‐week‐old female C57BL/6 mice.

### 2.14 Vectors construction and transduction

For DDIT4 overexpression, mouse *Ddit4* or human *DDIT4* fragment was amplified by PCR and subsequently subcloned into retroviral vector MSCV‐IRES‐RFP or lentiviral vector pCDH‐T2A‐GFP. Infectious retrovirus or lentivirus was prepared in 293T cells and collected at 48 and 72 h after transfection, and then, AE9a leukemia cells from established AE9a‐*Ddit4* ^−/−^ leukemia mice, as well as KG‐1a and Kasumi‐1 cells, were then transduced. Cells expressing GFP or RFP were sorted on FACS ArialII (BD Biosciences, San Jose, CA, USA) and utilized in subsequent experiments.

For re‐expression of Hoxa6, mouse *Hoxa6* was subcloned into retroviral vector MSCV‐IRES‐RFP (MSCV‐*Hoxa6‐*IRES‐RFP). AE9a leukemia cells were sorted from established AE9a‐*Ddit4* ^−/−^ leukemia mice and were then transfected with the prepared retrovirus.

### 2.15 CCK‐8 assay

Transfected Kasumi‐1 and KG‐1a cells were plated into 96‐well plates (1 × 10^4^ cells per well) and cultured for 24, 48, and 72 h. Ten microliters of CCK‐8 solution was added to each well and incubated at 37 °C for 4 h. Absorbance at 450 nm was subsequently measured. Each experimental condition was performed in triplicate and repeated three times.

### 2.16 Statistical analysis

Data were presented as mean ± SEM from triplicate experiments. Comparisons between two groups were determined using Student's unpaired *t*‐test. Survival curves were produced using the Kaplan–Meier estimates. *P* values < 0.05 were considered statistically significant differences.

## 3 Results

### 3.1 AE9a leukemia cells located in the endosteal region exhibit a transcriptional signature enriched in hypoxia and high *Ddit4* expression

**Fig. 1:**
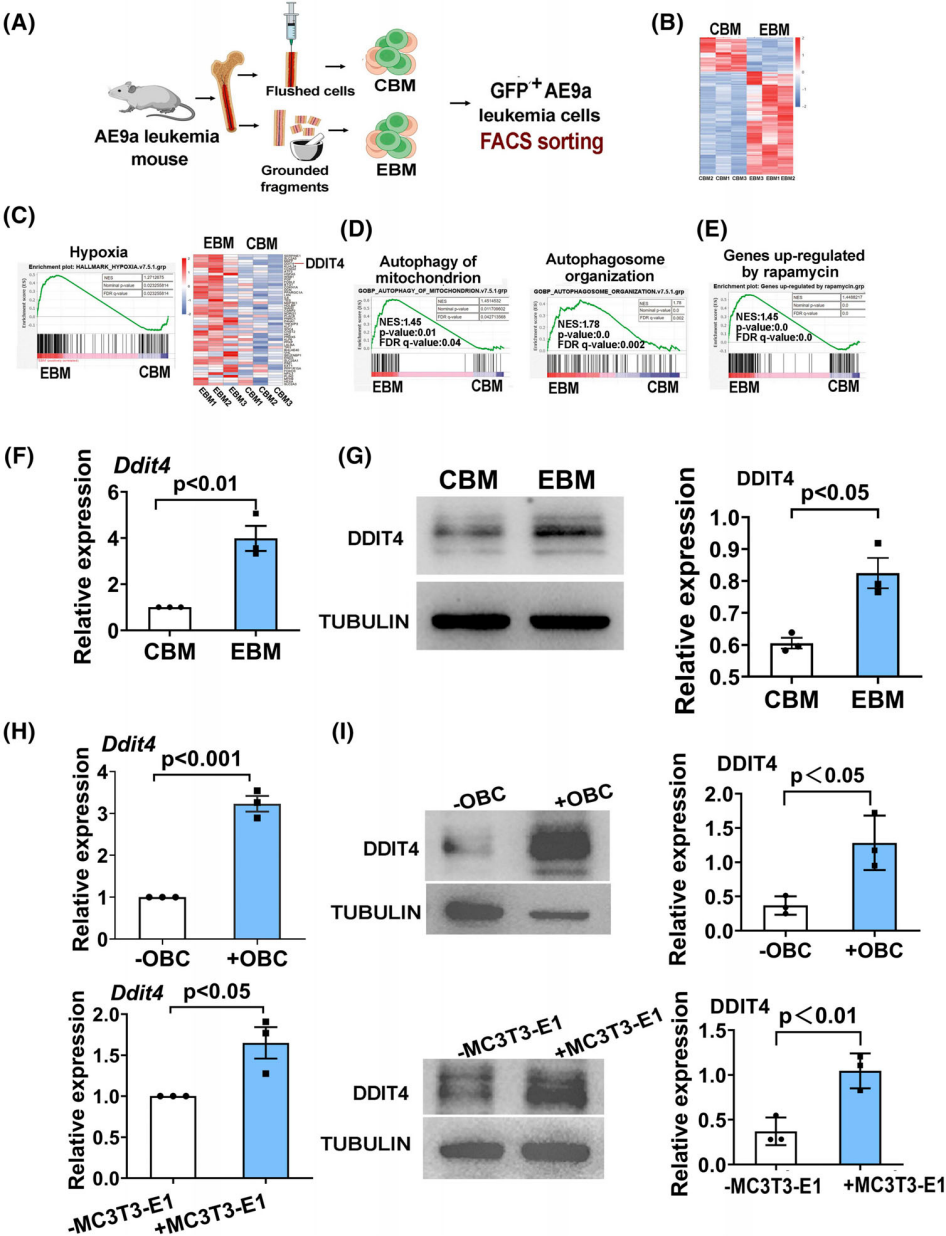
AE9a leukemia cells located in endosteal region display transcriptional response to hypoxia and upregulated *Ddit4* expression. CBM, central bone marrow; EBM, endosteal surface of bone marrow. OBC: mouse calvarial osteoblast. (A) Experimental scheme showing isolation of AE9a leukemia cells in CBM and EBM. (B) Heatmaps of the differentially expressed genes between CBM and EBM group. (C) Gene Set Enrichment Analysis (GSEA) plots showing enrichment of genes related to hypoxia in AE9a leukemia cell in EBM relative to that in CBM and the heat map of genes in hypoxia gene set. (D, E) GSEA plots of enriched genes involved in regulation response related to DDIT4, including autophagy of mitochondrion, autophagosome organization and genes upregulated by rapamycin. (F) qRT‐PCR analysis of *Ddit4* expression levels in AE9a leukemic cells isolated from CBM and EBM. *P* < 0.01. (G) Protein levels of DDIT4 in AE9a leukemia cells in AE9a leukemic cells isolated from CBM and EBM. *P* < 0.05. (H) qRT‐PCR analysis of relative expression of *Ddit4* in AE9a leukemia cells co‐cultured with mouse osteoblast cells and osteoblast cell line. Upper panel *P* < 0.001, Lower panel *P* < 0.05. (I) Protein levels of DDIT4 in AE9a leukemia cells co‐cultured with mouse osteoblast cells and osteoblast cell line. Left panel: representative western blot image of DDIT4 from three independent experiments. Right panel: relative protein levels of DDIT4. Upper panel *P* < 0.05, Lower panel *P* < 0.01. The values were calculated by the ratio of the band density of DDIT4 to that of internal control, and the band density was analyzed using Image J software (G, I). Data are presented as the mean ± SEM from three independent experiments (F–I). Each dot represents the value from an individual, with a total of three individuals included. The statistical significance were determined using Student's unpaired *t*‐test (F–I).

AML1‐ETO fusion gene, which is generated by the chromosomal translocation t(8;21)(q22;q22), is one of the most frequent fusion genes found in AML patients. An alternatively spliced form of AML1‐ETO, AML1‐ETO9a (AE9a), was identified in t(8;21) AML patient samples. This variant has been demonstrated to exhibit potent leukemogenic activity in mouse model, and this AE9a mouse model closely mimics AML patients initiated by AML1‐ETO [29]. Like normal HSCs, LSCs appear to reside in endosteal BM niche and utilize hypoxic niche for their stemness maintenance. To determine the molecular mechanism that regulating the stemness of leukemia cells, firstly, based on AE9a leukemia mouse model, we performed RNA‐seq analyses on leukemia cells isolated from collagenase treated bone (endosteal BM, EBM) and conventional flushed BM (central BM, CBM) of AE9a leukemia mice, respectively (Fig. 1A). AE9a cells in these two BM regions displayed different expression patterns (Fig. 1B). As expected, Gene Set Enrichment Analysis (GSEA) identified that highly expressed transcripts in endosteal AE9a cells were significantly enriched in the genes upregulated in response to hypoxia (Fig. 1C). Among the most differentially expressed genes in hypoxia response, it was noted that *Ddit4* was significantly more highly expressed in endosteal AE9a cells (Fig. 1C). As a negative regulator of mTOR, DDIT4 plays a key role in triggering autophagy under cellular stress. Therefore, we also analyzed the transcriptional signatures response to autophagy. For the differentially expressed transcripts in endosteal AE9a cells, significant enrichment of genes in GO biological process categories ‘Autophagy of Mitochondrion’ and ‘Autophagosome organization’ was noted (Fig. 1D). DDIT4 acts as a negative regulator of mTOR pathway in response to cellular stress, a mechanism consistent with the inhibitory effect of rapamycin on mTOR activity. Our analysis results indeed also showed that the genes upregulated in AE9a cells isolated from the endosteum displayed a transcriptional signature that was upregulated by rapamycin (Fig. 1E). The data indicate that the expression of DDIT4 could be induced and upregulated in AE9a leukemia cells located in the endosteal region. We further validated DDIT4 expression levels in leukemia cells isolated from the endosteal BM and central BM. As shown in Fig. 1F,G, the expression of DDIT4 in EBM AE9a cells was significantly higher than that in CBM AE9a cells, and this increase was identified at both mRNA and protein levels. These results are consistent with the DDIT4 expression patterns identified by RNA‐seq analysis.

To further confirm the effect of upregulated DDIT4 in AE9a leukemia cells by the endosteal region, we measured DDIT4 expression levels in AE9a cells co‐cultured with mouse OBC and BM MSC cells, as well as the mouse osteoblast cell line MC3T3‐E1 and MSC cell line OP9. The qRT‐PCR and western blot analyses showed that when co‐cultured with OBC and MC3T3‐E1 cells, AE9a cells had a significantly increased expression of DDIT4 (Fig. 1H,I), while there was no significant DDIT4 upregulation effect in AE9a cells when co‐cultured with MSC or OP9 cells (Fig. S1A,B). These results confirmed that in leukemia cells, DDIT4 could be induced in the endosteal BM and suggested that DDIT4 might participate in the stemness maintenance of leukemia cells in the hypoxic BM niche.

### 3.2 *DDIT4* expression identifies human AML patients with a transcriptional signature involved in hypoxia, quiescence, and LSC stemness

**Fig. 2:**
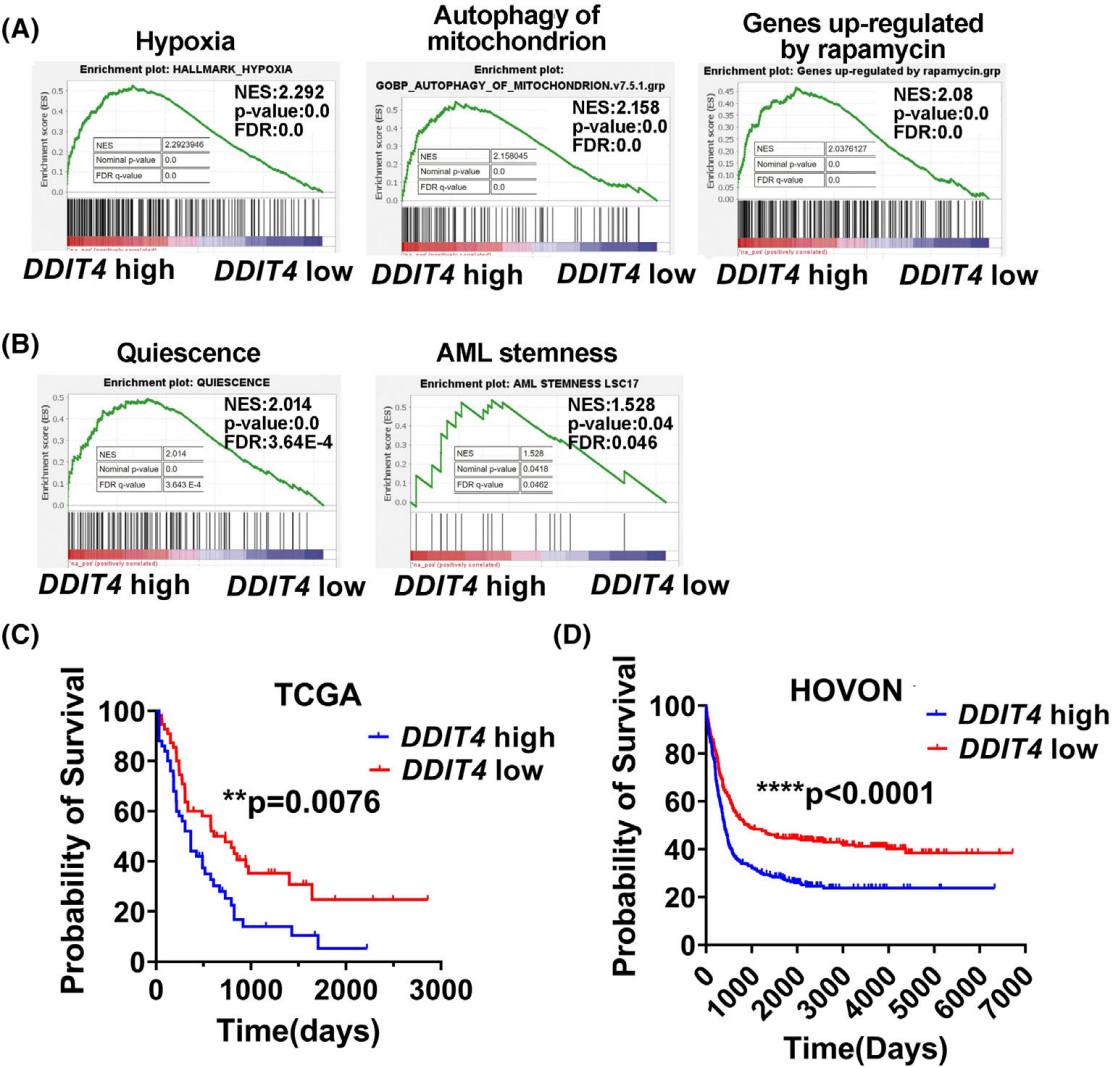
Gene Set Enrichment Analysis (GSEA) of transcriptional data and survival analysis of human adult acute myeloid leukemia (AML) patients based on *DDIT4* expression level. The gene expression profile and survival data of AML patients were downloaded from the TCGA database and the HOVON (E‐MTAB‐3444) dataset. Patients were assigned to high and low DDIT4 expression groups based on the median expression level. In TCGA AML cohort, high group: *n* = 56, low group: *n* = 57. In HOVON AML cohort, high group: *n* = 304, low group: *n* = 304. AML: acute myeloid leukemia. (A) GSEA plots showing enrichment of genes involved in hypoxia, autophagy of mitochondrion, and transcriptional signature that is upregulated by rapamycin in adult AML patients with high *DDIT4* expression (TCGA AML cohort). (B) Enrichment plot for quiescence and stemness related genes from GSEA of AML patients with high *DDIT4* expression (TCGA AML cohort). (C, D) Overall survival (OS) based on human *DDIT4* expression of adult leukemia patients from the TCGA AML cohort (C) and HOVON AML cohort (D). The statistical significance of survival was determined using the log‐rank (Mantel‐Cox) test (C, D). (C: *P* = 0.0076, D: *P* < 0.0001).

To demonstrate whether DDIT4 plays a role in human AML, we analyzed the data from the TCGA publicly available transcriptional database of adult AML patients. Based on this public database, a total of 113 AML patients were divided into two groups based on the median expression level of *DDIT4*, and the gene expression profile was compared between patients with high and low transcriptional expression of *DDIT4*. GSEA revealed that the high *DDIT4* expression group was highly enriched for hypoxia, autophagy of mitochondrion, and transcriptional signatures that were upregulated by rapamycin, which exhibited a relatively similar gene profile to mouse AE9a cells in the endosteal (Fig. 2A). These transcriptional signatures in the high *DDIT4* expression group were also validated in another adult AML patient cohort from the HOVON public database (Fig. S2). Specifically, patients with high *DDIT4* expression exhibited superiority in cell quiescence and LSC stemness (Fig. 2B). These data suggest that DDIT4 is not only associated with hypoxia but also modulates cell quiescence and stemness of LSCs in AML.

We then performed a Kaplan–Meier survival analysis with this TCGA AML cohort based on *DDIT4* expression levels. The overall survival time in AML patients with high *DDIT4* level (*n* = 57) was significantly shorter than that of those with low *DDIT4* expression (*n* = 56) (*P* < 0.01, Fig. 2C), indicating that a higher *DDIT4* level in leukemia cells is associated with worse prognosis. The association of overall survival with *DDIT4* expression was also validated in the AML patient cohort from the HOVON public database (*n* = 304 in each group, *P* < 0.0001, Fig. 2D). These help to support the hypothesis that DDIT4 may be involved in the chemoresistance and stemness of leukemia cells.

### 3.3 Leukemia cell stemness is highly dependent on DDIT4

**Fig. 3:**
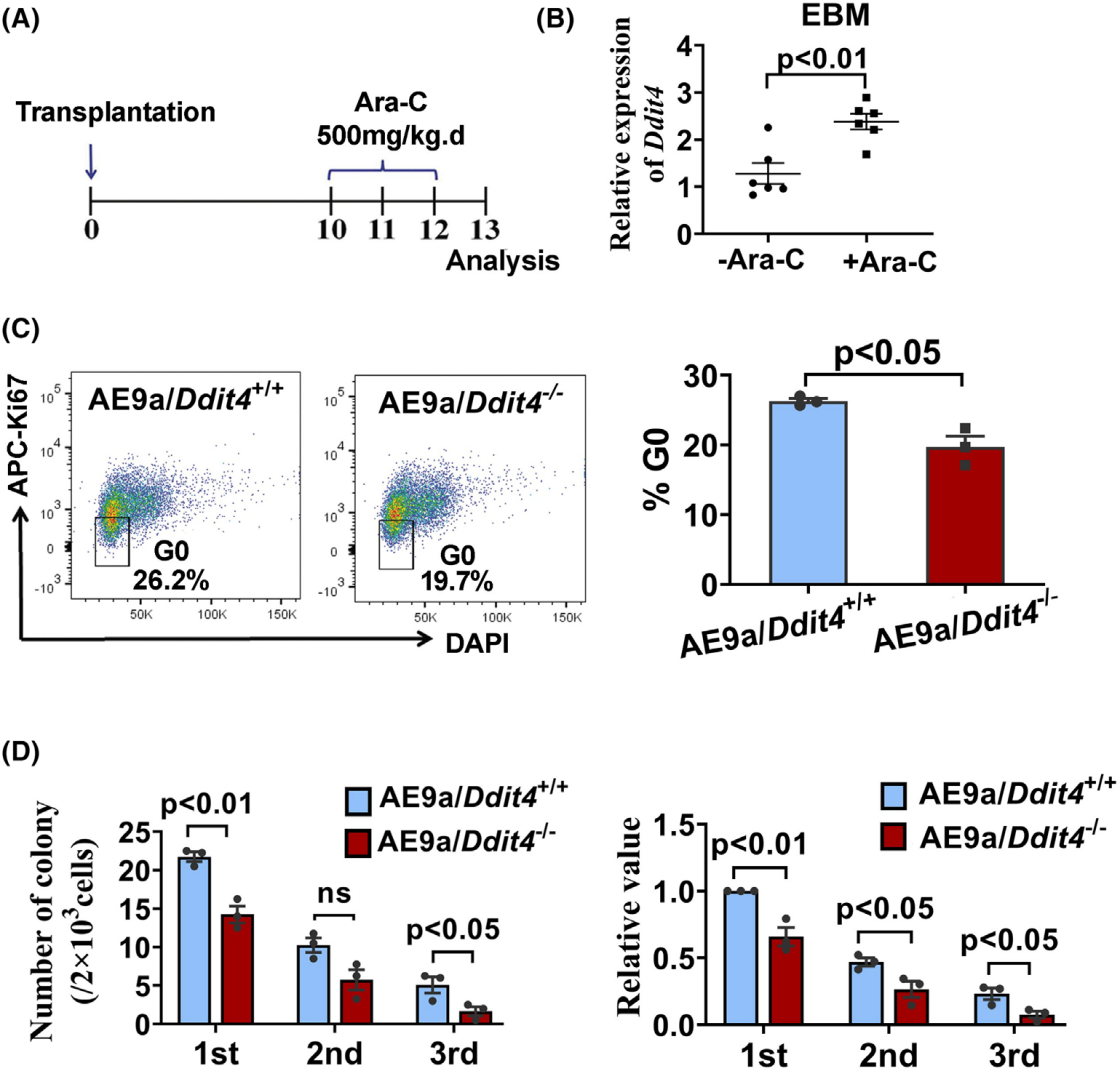
Chemoresistance of Ddit4 high‐expressing AE9a leukemia cells and reduction of G0 cell cycle phase and colony formation ability in AE9a expressed HSPCs of *Ddit4* deficient mice. (A) Ara‐C treatment regimen in AE9a leukemia mice. (B) Increased expression of *Ddit4* in AE9a cells in Ara‐C treated AE9a mice. Expression of *Ddit4* in AE9a cells in EBM from Ara‐C treated or untreated mice was determined by qRT‐PCR, and the data are presented as the mean ± SEM in one representative analysis (*n* = 6) of three independent experiments (*P* < 0.01). (C) G0 phase of the cell cycle analysis on AE9a transduced HSPCs from WT and *Ddit4* knockout mice. Left panel: Representative FACS plot for G0 phase analysis from three independent experiments. Right panel: The percentage of cells in G0 phase. Data are presented as the mean ± SEM from three independent experiments (*P* < 0.05). Each dot represents the relative value of the ratio of cells in G0 phase from one experiment. (D) Colony formation ability of AE9a transduced HSPCs from WT and *Ddit4* knockout mice. Left panel: Data are presented as the mean ± SEM from three independent experiments. Each dot represents the average absolute colony numbers per well (*n* = 4 in each group) from an individual, with a total of three individuals included. 1st: *P* < 0.01; 2nd: not significant; 3rd: *P* < 0.05. Right panel: Data are presented as the mean ± SEM from three independent experiments. Each dot represents the relative value of colony numbers divided by that of AE9a/*Ddit4* ^+/+^ group in the first round. (1st: *P* < 0.01; 2nd: *P* < 0.05; 3rd: *P* < 0.05). FACS sorted AE9a/*Ddit4* ^+/+^ or AE9a/*Ddit4* ^−/−^ cells were co‐cultured with MC3T3‐E1 osteoblast cells for 24 h, followed by G0 phase analysis and colony formation assays (C, D). The statistical significance was determined using Student's unpaired *t*‐test (B–D). HSPCs: hematopoietic stem and progenitor cells. EBM: endosteal surface of bone marrow.

In order to demonstrate the role of DDIT4 in leukemia cell stemness, we explored the function of DDIT4 in chemoresistance, quiescence, and self‐renewal of AE9a leukemia cells. Using an AE9a leukemia mouse model, we first determined whether *Ddit4* high‐expressing cells could resist chemotherapy. AE9a leukemia mice were treated with Ara‐C for 3 days, and then, *Ddit4* expression levels were determined in the EBM leukemia cells from Ara‐C treated and untreated mice (Fig. 3A). As shown in Fig. 3B, in Ara‐C treated AE9a leukemia mice, *Ddit4* expression in EBM leukemia cells was significantly higher than that in untreated mice. The median level in the Ara‐C treated group was 1.9‐fold higher than that in the untreated group, which indicated that high *Ddit4* expressing leukemia cells in EBM could be enriched by drug treatment. The chemo‐enrichment was not found in CBM leukemia cells (Fig. S3A), which might be due to the fact that high *Ddit4* expressing cells mainly located in EBM.

We next examined the role of DDIT4 in quiescence maintenance and self‐renewal of leukemia cells *in vitro*. Hematopoietic stem and progenitor cells (HSPCs) from *Ddit4* knockout (*Ddit4* ^−/−^) mice were used to evaluate the effect of *Ddit4* deletion on the properties of AE9a transfected HSPCs. c‐Kit^+^ cells from BM of *Ddit4* ^−/−^ and *Ddit4* ^+/+^ (WT) mice were transduced with AE9a by retroviral infection, respectively. Cell cycle analysis showed that there was no significant difference in the percentage of cells at G0 phase in GFP^+^ AE9a expression cells between AE9a/*Ddit4* ^+/+^ and AE9a/*Ddit4* ^−/−^ cells (Fig. S3B). Considering that due to the cells from *Ddit4* ^+/+^ and *Ddit4* ^−/−^ mice being cultured *in vitro* without the support of an *in vivo* environment, DDIT4 expression level in AE9a/*Ddit4* ^+/+^ cells may have declined, we then co‐cultured AE9a/*Ddit4* ^+/+^ and AE9a/*Ddit4* ^−/−^ cells with osteoblast cell MC3T3‐E1 for 24 h, respectively. After being co‐cultured with MC3T3‐E1, compared with AE9a/*Ddit4* ^+/+^ cells, AE9a/*Ddit4* ^−/−^ cells exhibited a significantly lower percentage of cells in G0 phase (Fig. 3C). This result indicates that *Ddit4* deletion leads to a decrease in the quiescence maintenance ability of AE9a expressing HSPCs. When cultured without MC3T3‐E1, AE9a/ *Ddit4* ^+/+^ cells exhibited a low DDIT4 expression level, and similar ratios of cells in G0 phase were found in AE9a/*Ddit4* ^+/+^ and AE9a/*Ddit4* ^−/−^ cells. Taken together, these results suggest that quiescence maintenance in AE9a transfected HSPCs is dependent on DDIT4 expression.

Furthermore, we performed a serial colony formation assay to determine the role of DDIT4 in self‐renewal *in vitro*. Under the conditions mentioned above, when precultured without MC3T3‐E1, AE9a/*Ddit4* ^−/−^ cells formed a lower number of colonies than those of AE9a/*Ddit4* ^+/+^ cells in the first round of the colony culture, but the difference was not significant (Fig. S3C). While co‐cultured with MC3T3‐E1, compared with AE9a/*Ddit4* ^+/+^ cells, AE9a/*Ddit4* ^−/−^ cells produced significantly fewer colonies in the first, second, and third rounds of the colony culture (Fig. 3D). AE9a/*Ddit4* ^−/−^ cells exhibited decreased re‐plating potential, indicating that *Ddit4* deletion attenuated self‐renewal capacity *in vitro*. Collectively, these results support the hypothesis that DDIT4 is essential for chemoresistance, quiescence, and self‐renewal of leukemia cells.

### 3.4 *Ddit4* deficiency leads to a defect in AE9a‐induced leukemia development

**Fig. 4:**
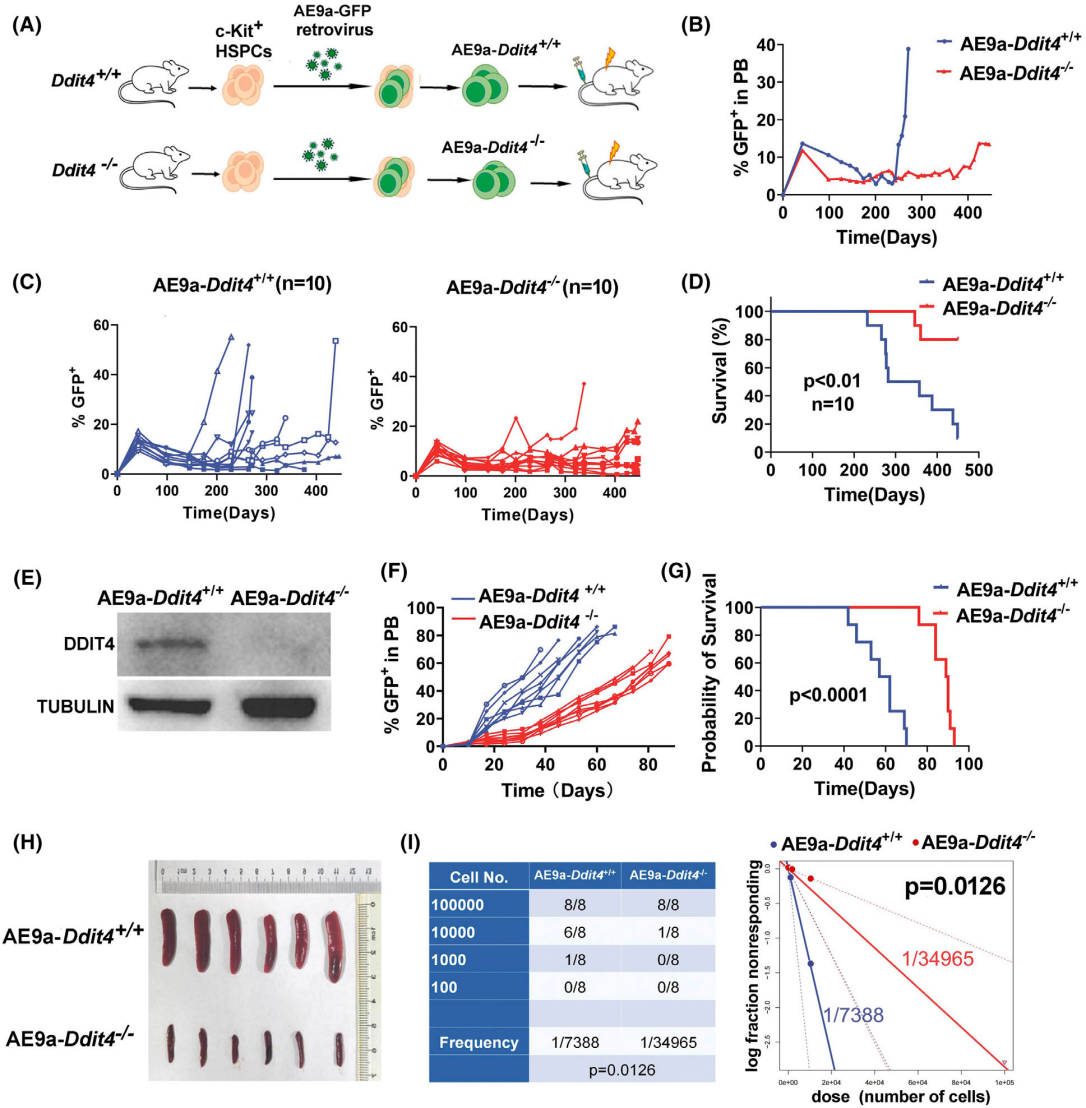
*Ddit4* deficiency results in a defect in AE9a‐induced leukemia initiation and development. (A) Experimental schema showing transplantation of AE9a retroviral infected HSPCs from *Ddit4* ^+/+^ and *Ddit4* ^−/−^ mice, respectively. (B) Dynamic monitoring of the percentage of GFP^+^ cells in the peripheral blood of mice transplanted with AE9a‐transfected HSPCs‐*Ddit4* ^+/+^ or HSPCs‐*Ddit4* ^−/−^. Circulating GFP^+^ cells in one representative mouse with the median survival time from each group are presented (*n* = 10 mice for each group). (C) Dynamic monitoring of the percentage of GFP^+^ cells in the peripheral blood of all mice transplanted with AE9a‐transfected HSPCs (*Ddit4* ^+/+^ or *Ddit4* ^−/−^) (*n* = 10 in each group). (D) Kaplan–Meier survival curves of recipient mice transplanted with AE9a‐transfected HSPCs from *Ddit4* ^+/+^ and *Ddit4* ^−/−^ mice (*n* = 10 mice for each group, *P* < 0.01). (E) Representative image of western blot analysis showing DDIT4 is deficient in AE9a‐*Ddit4* ^−/−^ leukemia cells from the transplanted mice that developed leukemia. The representative image was from three independent experiments with similar results. (F) Proportion of GFP^+^ cells in the peripheral blood of the secondary transplanted mice transplanted with AE9a‐*Ddit4* ^+/+^ and AE9a‐*Ddit4* ^−/−^ BM cells from the primary leukemia mice (*n* = 8 mice for each group). (G) Kaplan–Meier survival analysis of leukemia development of the secondary transplanted mice in AE9a‐*Ddit4* ^+/+^ and AE9a‐*Ddit4* ^−/−^groups (*n* = 8 mice for each group, *P* < 0.0001). A total of 5 × 10^5^ AE9a‐*Ddit4* ^+/+^ and AE9a‐*Ddit4* ^−/−^ leukemia cells, derived from the primary established leukemia mice (F0 generation), were transplanted into recipient mice, respectively (F, G). (H) Representative images of spleens from secondary transplanted mice in AE9a‐*Ddit4* ^+/+^ and AE9a‐*Ddit4* ^−/−^groups from three independent experiments with similar results. The spleens of the mice in two groups were separated and observed at the same time when AE9a‐ *Ddit4* ^+/+^ leukemia cells transplanted mice had a high circulating GFP^+^ cells (*n* = 6 mice for each group). (I) Reduced LSC frequency of AE9a‐*Ddit4* ^−/−^ leukemic cells. Limiting dilution transplantation experiments were performed with bone marrow cells from primary AE9a‐*Ddit4* ^+/+^ and AE9a‐*Ddit4* ^−/−^ cells transplanted leukemic mice. Left panel: different numbers of cells used for the transplantation and the number of mice that developed leukemia at each dose group. Right panel: graph shows the frequency of LSCs in AE9a‐*Ddit4* ^+/+^ and AE9a‐*Ddit4* ^−/−^ leukemic cells (*P* = 0.0126). The frequency of the LSCs was calculated using the ELDA software. The statistical significance was determined by a likelihood ratio test based on the Poisson limiting dilution model in ELDA software. The statistical significance of survival was determined using the log‐rank (Mantel‐Cox) test (D, G). HSPCs: hematopoietic stem and progenitor cells. BM, bone marrow; LSCs, leukemia stem cells.

Self‐renewal is critical for leukemia initiation. To elucidate the role of DDIT4 in the self‐renewal of leukemia cells, we further examined the effect of deletion of *Ddit4* in AE9a‐induced leukemogenesis. HSPCs derived from *Ddit4* ^+/+^ and *Ddit4* ^−/−^ mice were used to establish AE9a‐induced leukemia models. c‐Kit^+^ cells sorted from BM of *Ddit4* ^+/+^ and *Ddit4* ^−/−^ mice were transfected with AE9a, respectively, then equal numbers of GFP^+^ cells in AE9a‐*Ddit4* ^+/+^ and AE9a‐*Ddit4* ^−/−^ groups were transplanted into lethally irradiated recipient mice (Fig. 4A). The percentage of GFP^+^ cells in the peripheral blood (PB) of each mouse was monitored for 15 months. The percentages of monitored GFP^+^ cells in PB of the representative mouse with the median survival time in each group were shown in Fig. 4B. As shown in Fig. 4C, elevated circulating GFP^+^ cells in PB (41%) were observed from day 201 after transplantation (range: day 201 to > day 389; median: days 389) in the AE9a‐*Ddit4* ^+/+^ group (*n* = 10). Following a rapid increase in GFP^+^ cells, AE9a‐*Ddit4* ^+/+^ mice exhibited a more immature myeloid phenotype and subsequently progressed to leukemia (Fig. S4A). However, in the AE9a‐*Ddit4* ^−/−^ group (*n* = 10), the proportion of GFP^+^ cells in PB remained consistently at a low level, and a marked elevation was not observed until day 338 (range: Day 338 to > Day 450, median: > Day 450) after transplantation (Fig. 4C). Until 450 days, 9 of 10 mice developed leukemia in the AE9a‐*Ddit4* ^+/+^ group, while in the AE9a‐*Ddit4* ^−/−^ group, only 2 of 10 mice developed leukemia with a similar pathology and immunophenotype to AE9a‐*Ddit4* ^+/+^ mice (Fig. S4B). Survival analysis showed that the median survival time of AE9a‐induced leukemia was significantly prolonged by deletion of *Ddit4* (Fig. 4D). Western blot analysis confirmed the complete lack of DDIT4 expression in AE9a‐*Ddit4* ^−/−^ leukemia cells (Fig. 4E). These results demonstrate that DDIT4 deficiency can significantly impede AE9a‐induced leukemia development and suggest that DDIT4 takes an important role in leukemia initiation.

To further assess whether DDIT4 is necessary for AML leukemia maintenance, we performed secondary transplantation. The same numbers of AE9a‐*Ddit4* ^+/+^ and AE9a‐ *Ddit4* ^−/−^ GFP^+^ leukemia cells were transplanted into sublethally irradiated recipients, respectively. As shown in Fig. 4F, after transplantation, circulating GFP^+^ cells in the AE9a‐*Ddit4* ^+/+^ group were higher than those in the AE9a‐*Ddit*4^−/−^ group at each time point. In the observation of survival, in the AE9a‐*Ddit4* ^+/+^ group, all recipient mice developed leukemia with a median survival time of 59.5 days, and all mice died before Day 70, whereas at that time point, in the AE9a‐*Ddit4* ^−/−^ group, none of the mice developed leukemia. The mice in the AE9a‐*Ddit4* ^−/−^ group developed leukemia with a median latency of 89.5 days. AE9a‐*Ddit4* ^−/−^ mice displayed significantly extended latency compared with AE9a‐*Ddit4* ^+/+^ mice (Fig. 4G). Once a mouse transplanted with AE9a‐*Ddit4* ^+/+^ leukemia cells showed a high circulating GFP^+^ cell count (defined as > 80% GFP^+^ cells in PB), all six recipient mice in each group (total 12 mice) were euthanized simultaneously. The mice in the *Ddit4* ^+/+^ group displayed a severe splenomegaly, while the AE9a‐*Ddit4* ^−/−^ cell transplanted mice did not (Fig. 4H). Deletion of *Ddit4* resulted in delayed AE9a‐induced leukemia development, indicating that DDIT4 is critical for leukemia development and the self‐renewal capacity of leukemia cells.

Furthermore, using AE9a‐*Ddit4* ^+/+^ and AE9a‐*Ddit4* ^−/−^ leukemia cells from the primary transplanted mice, the LSC frequency and the role of DDIT4 in self‐renewal of leukemia cells were further identified via limiting dilution transplantation assay. As expected, six out of eight mice that received 10 000 AE9a‐*Ddit4* ^+/+^GFP^+^ cells developed leukemia with a mortality of 75%, while the mortality of mice receiving 10 000 AE9a‐*Ddit4* ^−/−^ GFP^+^ leukemic cells was only 12.5%. The leukemia initiating frequency in AE9a‐*Ddit4* ^−/−^ leukemic cells was 4.7‐fold lower than that in AE9a‐*Ddit4* ^+/+^ leukemic cells (1/34965 versus 1/7388 cells, *P* = 0.0126, Fig. 4I). This result indicates that deletion of *Ddit4* markedly reduced the LSC frequency, further confirming that DDIT4 is critical in self‐renewal and leukemia initiation of leukemia cells.

**Fig. 5:**
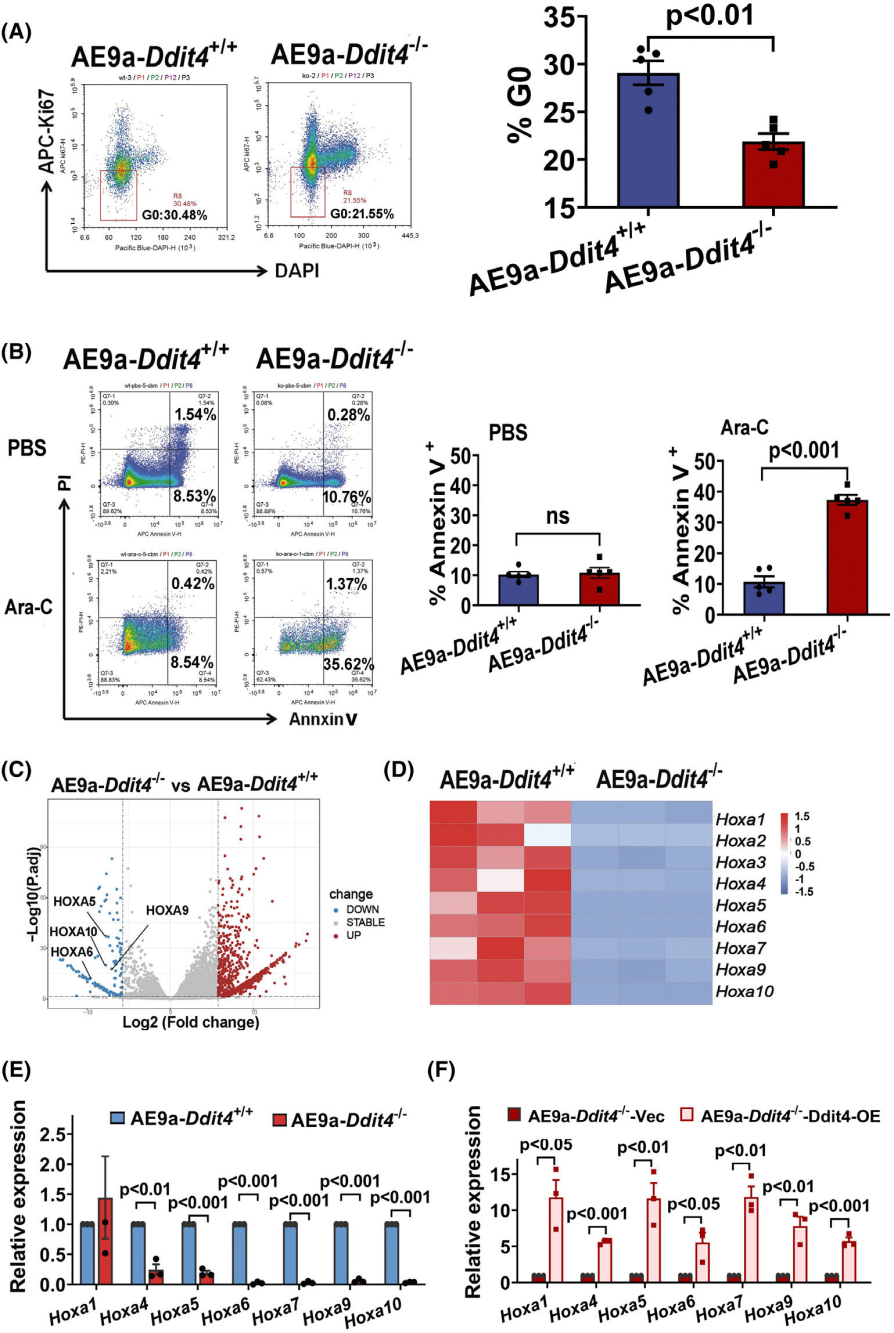
Reduced proportion of cells in the G0 phase, decreased chemoresistance, and downregulated expression of Hoxa cluster genes in AE9a‐*Ddit4* ^−/−^ leukemia cells. (A) Proportion of cells in the G0 phase in AE9a‐*Ddit4* ^+/+^ and AE9a‐*Ddit4* ^−/−^ leukemia cells. Leukemia cells were isolated from the BM niche of AE9a‐*Ddit4* ^+/+^ and AE9a‐*Ddit4* ^−/−^. Left panel: Representative FACS plot for G0 phase analysis. Right panel: The percentage of cells in the G0 phase in GFP^+^ leukemia cells in each group (*n* = 5 mice for each experiment from three independent experiments, *P* < 0.01). (B) Apoptosis analysis of leukemia cells from Ara‐C treated AE9a‐*Ddit4* ^−/−^ and AE9a‐*Ddit4* ^+/+^ leukemia mice. When the circulating GFP^+^ cells reached 10–20% after transplantation, the mice were treated with 500 mg·kg^−1^ Ara‐C for 3 days, and then, apoptosis was evaluated by FACS analysis following Annexin V and PI staining. Left panel: Representative FACS plots of apoptosis analysis indicated by the percentage of Annexin V^+^ cells in GFP^+^ leukemia cells from Ara‐C treated AE9a‐*Ddit4* ^−/−^ and AE9a‐*Ddit4* ^+/+^ leukemia mice. Right panel: The values represent the proportions of Annexin V^+^ cells in GFP^+^ leukemia cells in each group (*n* = 5 mice for each experiment from three independent experiments. PBS: not significant, Ara‐C: *P* < 0.001). (C) Volcano plot showing adjusted *P* value (Log10 padj) vs Log_2_ fold changes of each gene tested between AE9a‐*Ddit4* ^−/−^ and AE9a‐*Ddit4* ^+/+^ leukemia cells from RNA sequencing data. (D). Heatmap of differentially expressed Hoxa cluster genes of AE9a‐*Ddit4* ^+/+^ vs AE9a‐*Ddit4* ^−/−^ leukemia cells. The cells were obtained from the BM of the mice transplanted with AE9a‐*Ddit4* ^+/+^ and AE9a‐*Ddit4* ^−/−^ cells (*n* = 3 in each group). (E). Downregulated Hoxa cluster genes in AE9a‐*Ddit4* ^−/−^ cells confirmed by real‐time RT‐PCR. (F). Upregulated Hoxa cluster genes expression by re‐expression of Ddit4 in AE9a‐*Ddit4* ^−/−^ leukemia cells. The data are presented as the mean ± SEM from three independent experiments, and each dot represents the value from an individual, with a total of three individuals included (E, F). Exact *P* values for all pairwise comparisons are annotated above their corresponding bars (E, F). The statistical significance was determined using Student's unpaired *t*‐test (A, B, E, F). BM, bone marrow.

In addition to its role in self‐renewal, we further investigated the involvement of DDIT4 in quiescence and chemotherapy resistance using AE9a‐*Ddit4* ^−/−^ and AE9a‐*Ddit4* ^+/+^ leukemia cells isolated from the mice and analyzed *ex vivo*. Cell cycle analysis revealed a significantly lower proportion of cells in the G0 phase in leukemia cells isolated from EBM of AE9a‐*Ddit4* ^−/−^ mice when compared to that of AE‐9a‐*Ddit4* ^+/+^ mice (Fig. 5A). Furthermore, to assess chemoresistance, AE9a‐*Ddit4* ^−/−^ and AE9a‐*Ddit4* ^+/+^ mice were treated with Ara‐C for 3 days *in vivo*. As shown in Fig. 5B, AE9a‐*Ddit4* ^−/−^ cells displayed a significantly higher proportion of Annexin V‐positive cells than AE9a‐*Ddit4* ^+/+^ cells (*P* < 0.001), indicating that DDIT4 deficiency increases cell sensitivity to chemotherapy. The results demonstrate that DDIT4 plays a critical role in maintaining quiescence and chemoresistance in AE9a leukemia cells *in vivo*.

### 3.5 *Ddit4* deficiency results in downregulated Hoxa cluster expression, which can be recovered by overexpression of *Ddit4* in leukemia cells

To better understand the molecular mechanisms underlying the role of DDIT4 in self‐renewal of leukemia cells and leukemia initiation, we performed global gene expression profiling by RNA sequencing on AE9a‐*Ddit4* ^+/+^ and AE9a‐*Ddit4* ^−/−^ leukemia cells. RNA sequencing revealed that AE9a cells from these two groups exhibited distinct transcriptional profiles. *Ddit4* depletion led to a downregulation on 2053 genes and upregulation on 4883 genes. Among the downregulated genes, it could be found that several genes related to self‐renewal potential were significantly downregulated, including *Hoxa1, Hoxa2, Hoxa3, Hoxa4, Hoxa5, Hoxa6, Hoxa7, Hoxa9*, and *Hoxa10* (Fig. 5C,D). Correspondingly, significantly lower expression of *Hoxa4, Hoxa5, Hoxa6, Hoxa7, Hoxa9*, and *Hoxa10* in AE9a‐*Ddit4* ^−/−^ cells were confirmed by real‐time RT‐PCR (Fig. 5E). These data suggest that DDIT4 may upregulate *Hoxa* cluster genes. To achieve this, we re‐expressed *Ddit4* in AE9a‐*Ddit4* ^−/−^ leukemic cells. Conversely, the significant increases in *Hoxa* cluster gene expression were observed after *Ddit4* re‐expression in AE9a‐*Ddit4* ^−/−^ leukemic cells (Fig. 5F).

**Fig. 6:**
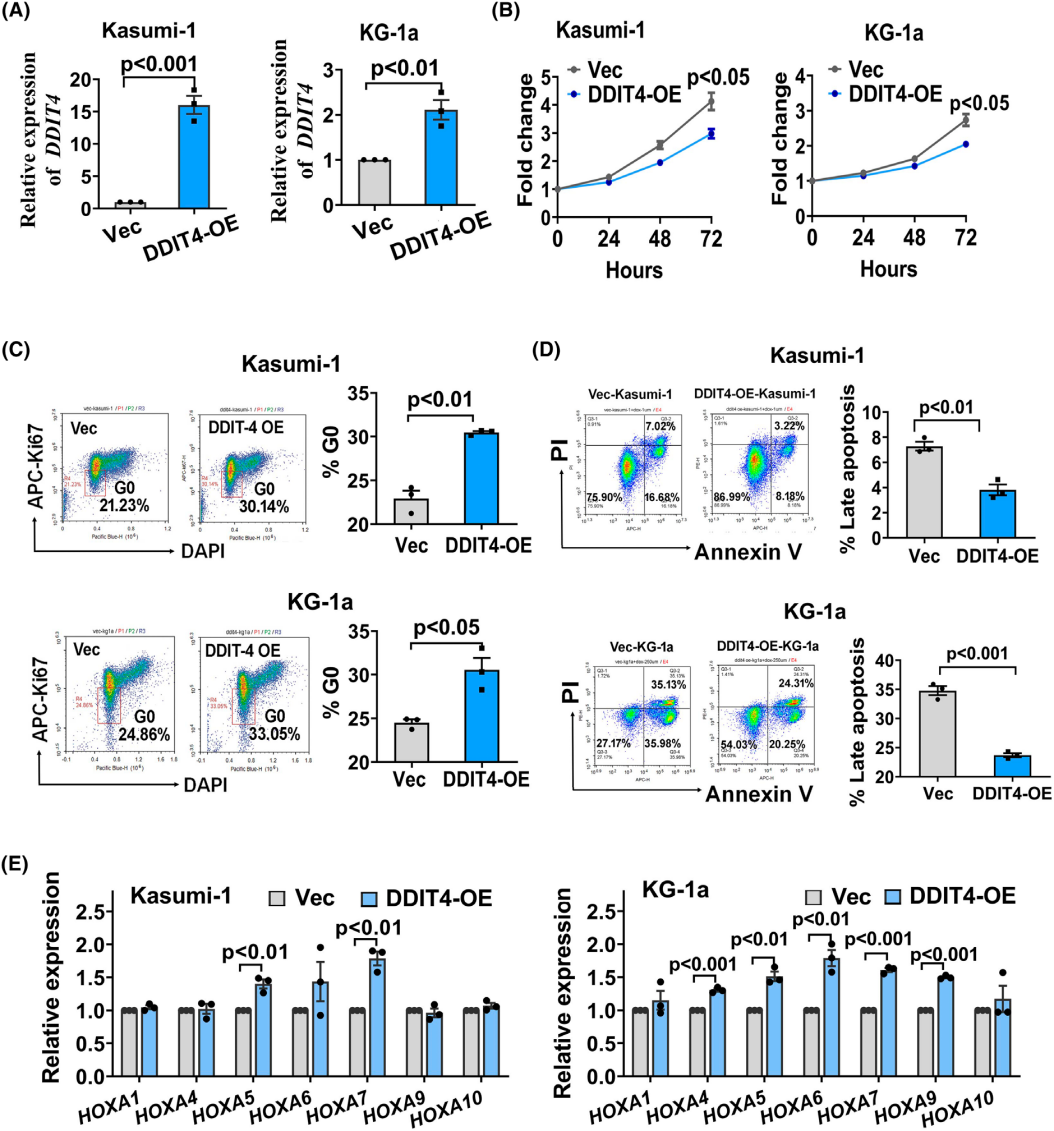
Overexpression of DDIT4 increases the ratio of cells in the G0 phase and chemoresistance, and upregulates Hoxa cluster gene expression in leukemia cells. (A) Forced expression of *DDIT4* in Kasumi‐1 and KG‐1a leukemia cell lines. (B) Decreased proliferative capacity upon DDIT4 overexpression in leukemia cell lines. Cell proliferation was determined by CCK‐8 assay. (C) Cell cycle analysis upon DDIT4 overexpression in leukemia cell lines. Left panel: Representative FACS plots for G0 phase analysis from three independent experiments. Right panel: The percentage of cells in G0 phase. Kasumi‐1: *P* < 0.01; KG‐1a: *P* < 0.05. (D) Analysis of apoptosis levels in Kasumi‐1 and KG‐1a cells under DDIT4 overexpression when treated with DNR. The Kasumi‐1 cells and KG‐1a cells were treated with 1 and 2.5 μm DNR, respectively, for 24 h, followed by apoptosis analysis. Left panel: Representative FACS plots for apoptosis analysis based on PI and Annexin V‐APC labeling. The representative plots were from three independent experiments. Right panel: The percentage of Annexin V^+^PI^+^ cells. Kasumi‐1: *P* < 0.01; KG‐1a: *P* < 0.001. (E) Upregulated *HOXA* cluster gene expressions upon DDIT4 overexpression in leukemia cell lines. Exact *P* values for all pairwise comparisons are annotated above their corresponding bars. Expression of *Hoxa* cluster genes or *DDIT4* was determined by qRT‐PCR (A, E). The data are presented as the mean ± SEM from three independent experiments. The statistical significance were determined using Student's unpaired *t*‐test (A–E). Each dot represents the value of one experiment from three independent experiments. (A, C–E). DNR, daunorubicin; PI, propidium iodide.

Additionally, DDIT4 was also forced expressed in Kasumi‐1 and KG‐1a human AML cell lines (Fig. 6A). For the observation on cell growth, it was found that both cell lines displayed slightly decreased proliferative capacity upon DDIT4 overexpression; however, spontaneous apoptosis was not observed in DDIT4‐overexpressed cells (Fig. 6B, Fig. S5). Cell cycle analysis revealed that in both cell lines, in DDIT4 overexpression cells, the ratio of cells in the G0 cell cycle phase was increased significantly (Fig. 6C), indicating that DDIT4 plays a role in maintaining quiescence in leukemia cells. Additionally, the role of DDIT4 in chemotherapy resistance was evaluated using DDIT4‐overexpressed Kasumi‐1 and KG‐1a cells treated with Daunorubicin (DNR). As shown in Fig. 6D, when treated with 1 μm DNR, the DDIT4‐overexpressed Kasumi‐1 cells had significantly lower apoptosis levels compared with Vec‐Kasumi‐1 cells (*P* < 0.01). Because KG‐1a cells had a higher IC50 for Daunorubicin than Kasumi‐1 cells, a similar pattern of chemoresistance was observed in DDIT4‐overexpressed KG‐1a cells when treated with 2.5 μm DNR. These findings indicate that overexpression of DDIT4 confers chemoresistance to leukemia cells.

The expression levels of *HOXA* cluster genes in response to DDIT4 overexpression were subsequently analyzed. Notably, DDIT4 overexpression significantly upregulated *HOXA* cluster gene expressions in both kasumi‐1 and KG‐1a cells (Fig. 6E), confirming DDIT4 can upregulate *HOXA* gene expressions in leukemia cells.

### 3.6 Leukemia initiation defect in AE9a‐*Ddit4* ^−/−^ cells can be rescued by re‐expression of Hoxa6

**Fig. 7:**
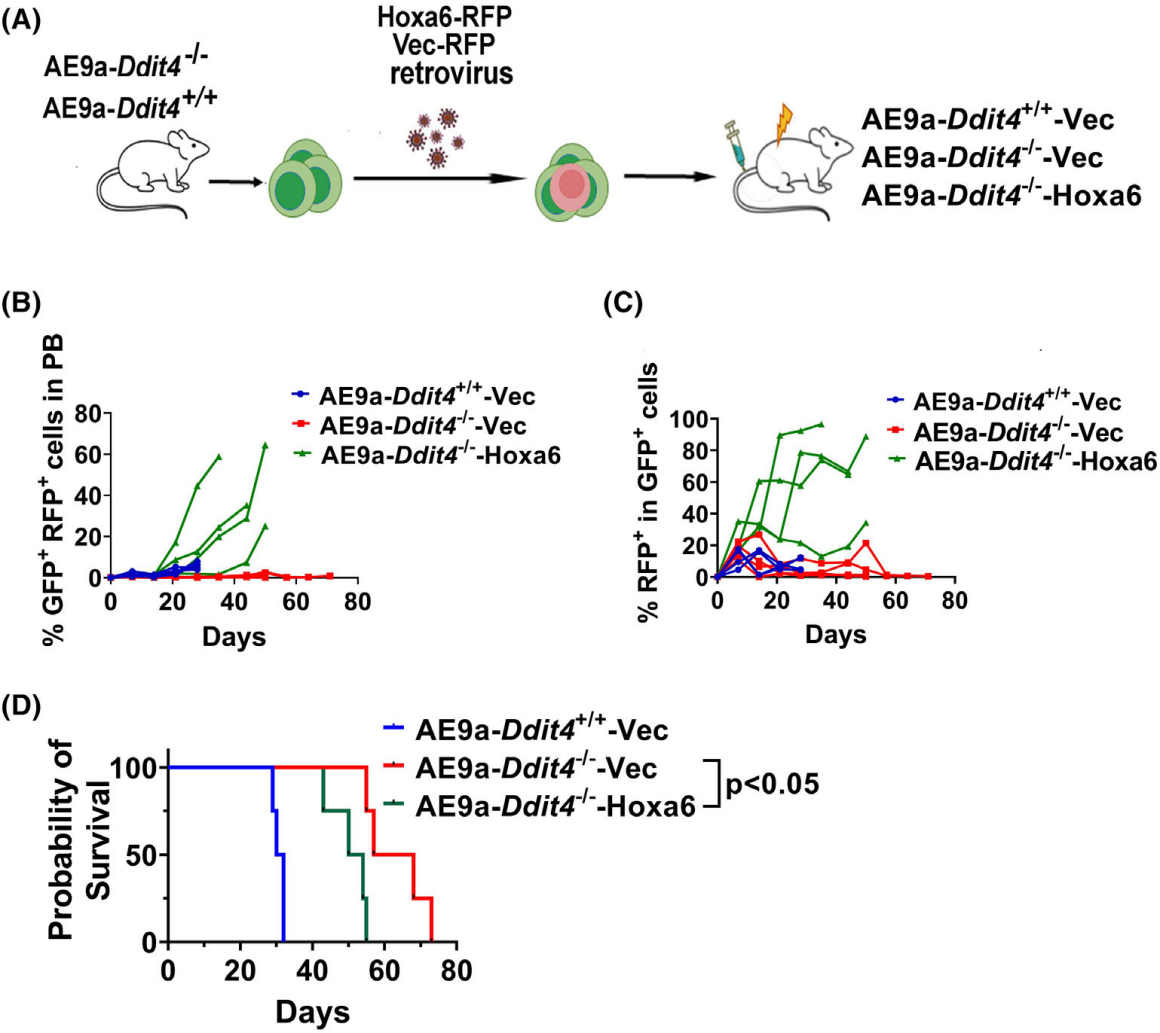
Defect in AE9a‐induced leukemogenesis caused by Ddit4 deletion can be rescued by re‐expression of Hoxa6. AE9a leukemia cells were obtained from the secondary transplanted AE9a‐*Ddit4* ^−/−^ or AE9a‐*Ddit4* ^+/+^ leukemia mice (F1 generation) and subsequently transduced with either MSCV‐*Hoxa6‐*IRES‐RFP or MSCV‐RFP (Vec). For transplantation, an equal number of GFP^+^RFP^+^ cells (8 × 10^4^) and GFP^+^ cells (1 × 10^6^) were injected into lethally irradiated (9Gy) female C57BL/6 mice in each group. RFP^+^GFP^+^ cells represent AE9a cells expressing Hoxa6 in the AE9a‐*Ddit4* ^−/−^‐Hoxa6 group. (A). Experimental schema showing mice transplanted with *Hoxa6*‐RFP retroviral infected AE‐*Ddit4* ^−/−^ leukemia cells. (B, C) Dynamic monitoring of the percentage of RFP^+^GFP^+^ cells (B) or percentage of RFP^+^ in GFP^+^ (C) in the peripheral blood of AE9a‐*Ddit4* ^−/−^‐Hoxa6 cells, AE9a‐*Ddit4* ^−/−^‐Vec cells, or AE9a‐*Ddit4* ^+/+^‐Vec cells transplanted mice (*n* = 4 in each group). (D) Kaplan–Meier survival curves of recipient mice transplanted with AE9a‐*Ddit4* ^−/−^‐Hoxa6 cells, AE9a‐*Ddit4* ^−/−^‐Vec cells, or AE9a‐*Ddit4* ^+/+^‐Vec cells (*n* = 4 in each group). The statistical significance of survival was determined using the log‐rank (Mantel‐Cox) test.

*Ddit4* deletion resulted in a defect in AE9a‐induced leukemogenesis and caused downregulated *Hoxa* cluster gene expression, suggesting that decreased *Hoxa* cluster expression confers a leukemia initiation defect in AE9a‐*Ddit4* ^−/−^ cells. Given that *Hoxa6* was the most significantly downregulated *Hoxa* cluster gene in AE9a‐*Ddit4* ^−/−^ cells and had been demonstrated to induce malignant transformation of primary hematopoietic cells [30], we next investigated whether re‐expression of *Hoxa6* could rescue the attenuated leukemia initiation potential caused by *Ddit4* deletion. AE9a‐*Ddit4* ^−/−^ GFP^+^ leukemia cells were retrovirally transduced with *Hoxa6*‐RFP expressing construct (MSCV‐*Hoxa6‐*IRES‐RFP) or empty vector (MSCV‐IRES‐RFP). Equal number of GFP^+^RFP^+^ cells (8 × 10^4^) in each group were transplanted into lethally irradiated recipient mice (Fig. 7A).

For the monitoring of RFP^+^GFP^+^ cells in each mouse, elevated circulating RFP^+^ GFP^+^ cells were observed in AE9a‐*Ddit4* ^−/−^‐*Hoxa6* mice on day 20–50 after transplantation, while all mice in the AE9a‐Ddit4^−/−^Vec group displayed no detectable RFP fluorescence until day 71 (Fig. 7B). It was also observed that the percentage of RFP^+^ cells in GFP^+^ leukemic cells was higher in the AE9a‐*Ddit4* ^−/−^‐*Hoxa6* group than that in the AE9a‐*Ddit4* ^−/−^Vec group at each time point (Fig. 7C). The proportion of RFP^+^ cells within the GFP^+^ leukemic cell population significantly increased in the AE9a‐*Ddit4* ^−/−^‐*Hoxa6* group, whereas it remained consistently low in the AE9a‐*Ddit4* ^−/−^‐Vec group, indicating that the expression of *Hoxa6* might confer a growth advantage in AE9a‐*Ddit4* ^−/−^ leukemia cells.

For the observation on leukemia development and survival of the mice, AE9a‐*Ddit4* ^+/+^Vec cell‐transplanted mice developed leukemia with a median survival of 31 days, and the median survival in the AE9a‐*Ddit4* ^−/−^Vec group was 62.5 days (Fig. 7D). As expected, AE9a‐*Ddit4* ^−/−^ *Hoxa6* cell‐transplanted mice developed leukemia with a significantly shorter survival time (median survival: 53 days) compared with AE9a‐*Ddit4* ^−/−^Vec mice (Fig. 7D), indicating that re‐expression of *Hoxa6* in AE9a‐*Ddit4* ^−/−^ cells accelerated leukemia development. It was confirmed that re‐expression of *Hoxa6* can partly rescue the leukemia initiation defect caused by *Ddit4* deletion. Taken together, it suggests that the critical role of DDIT4 in leukemogenesis is mediated by the HOXA cluster.

## 4 Discussion

Here, we demonstrate that DDIT4 can be upregulated in the endosteal bone marrow region in the AE9a leukemia mouse, and *Ddit4* deficiency suppresses AML initiation and leads to a defect in quiescence maintenance and self‐renewal of leukemia cells. Analysis of the AML cohort from the public database demonstrates that patients with high DDIT4 expression have shorter survival times, implying that DDIT4 may be involved in the chemoresistance of leukemia cells. Our study provides evidence for the critical role of DDIT4 in leukemia development and stemness maintenance of LSCs.

Contribution of the BM microenvironment, especially the endosteal niche, to leukemia development and LSCs chemoresistance has been well established [10, 24, 31]; however, intrinsic molecular alterations in leukemia cells caused by the BM niche and how the alterations facilitate the stemness maintenance of LSCs remain to be fully elucidated. Here, we found that DDIT4 in leukemia cells could be upregulated in the endosteal niche and DDIT4 was critical for leukemia cells stemness maintenance. In our *in vitro* assay, it was found that compared with AE9a/*Ddit4* ^−/−^ cells, AE9a/*Ddit4* ^+/+^ leukemia cells did not exhibit significant differences in colony formation and G0 cell cycle phase under the condition that the cells were cultured without osteoblast cells. However, after these leukemia cells were co‐cultured with osteoblast cells, AE9a/*Ddit4* ^+/+^ cells exhibited a significant advantage in colony formation ability and had more cells in G0 phase. These results can be explained by the fact that in the absence of osteoblast cells, AE9a/*Ddit4* ^+/+^ cells have a low expression level of DDIT4, and AE9a/*Ddit4* ^+/+^ cells could not exhibit increased stemness compared with AE9a/*Ddit4* ^−/−^ cells. It makes it more believable that in leukemia cells *in vivo*, DDIT4 can be induced by the endosteal niche and perform its function.

We also observed that when AE9a/*Ddit4* ^+/+^ or AE9a/*Ddit4* ^−/−^ cells were co‐cultured with osteoblast cells using Transwell plates, which prevented direct cell–cell contact, AE9a/*Ddit4* ^−/−^ cells showed no significant difference in the percentage of G0 phase cells compared with AE9a/*Ddit4* ^+/+^ cells (Fig. S6A,B). These results suggest that DDIT4‐mediated stemness maintenance requires direct interaction between AE9a cells and osteoblast cells. This conclusion was further supported by quantitative analysis showing that *Ddit4* expression in AE9a/*Ddit4* ^+/+^ cells under direct co‐culture conditions reached 2.65 times the level observed in AE9a/*Ddit4* ^+/+^ cells cultured alone, whereas under Transwell‐based co‐culture conditions, it was only 1.28 times. These data demonstrate that *Ddit4* upregulation is primarily dependent on direct cell–cell contact rather than osteoblast‐secreted factors. Collectively, our findings demonstrate that the stemness properties regulated by DDIT4 are mediated through direct interaction between AE9a cells and the endosteal niche.

In addition, our RNA‐seq data displayed that DDIT4 expression was closely associated with hypoxia. The BM endosteal region is a hypoxic environment; it is reasonable to assume that hypoxia is also important for DDIT4 induction. We also evaluated the DDIT4 expression in AE9a cells under three conditions: hypoxia alone, osteoblast co‐culture alone, and hypoxia combined with osteoblast co‐culture. The results demonstrated that hypoxia alone can induce an increase in DDIT4 expression in AE9a cells. Under hypoxic conditions combined with osteoblast co‐culture, DDIT4 expression levels did not show further increase than osteoblast co‐culture alone (Fig. S7A,B). This observation suggests that in the endosteal BM region, both hypoxia and osteoblast contact independently upregulate DDIT4 expression, but a synergistic effect between these two factors may not exist.

As a negative regulator of the mTOR kinase complex 1, DDIT4 can be induced under the stress conditions, such as chemotherapy, hypoxia, and endoplasmic reticulum stress [32, 33, 34]. It has been identified that a high expression level of DDIT4 is correlated with poor prognosis in multiple cancer types, including AML [21, 22, 35]. In a recent study utilizing TCGA and GTEx public databases, Li et al. demonstrated that high DDIT4 expression in AML patients was associated with poor prognosis. Furthermore, the knockdown of DDIT4 was shown to promote cell apoptosis in leukemia cell lines [36]. Under normal conditions, mTOR is important for tumor propagation and progression. However, under the stress conditions especially chemotherapy, DDIT4 is more important for tumor survival and cancer stem cell maintenance [22, 37, 38]. This is likely to explain why there is an insignificant or even negative correlation between DDIT4 overexpression and poor prognosis in some types or subtypes of tumors [21, 22, 39]. For example, in breast cancer, DDIT4 overexpression was significantly related to a poor prognosis in the basal subtype, while there is no such correlation in the HER2‐enriched subtype. Moreover, it was shown that high DDIT4 expression predicted poor prognosis only in AML patients undergoing chemotherapy, but not in the patients treated with allogeneic hematopoietic stem cell transplantation [21].

Self‐renewal potential of LSC is critical not only for leukemia initiation but also for disease recurrence after chemotherapy, which is related to disease relapse [3, 4]. Here, we show that DDIT4 is critical for self‐renewal and chemoresistance, suggesting that DDIT4 plays an important role in the maintenance of the LSC pool and is responsible for relapse. It has been reported that DDIT4 can mediate cellular adaptive therapy resistance in glioblastoma [37]. Although the antiapoptotic effect of DDIT4 has been preliminarily demonstrated in leukemia cells [36, 40], the underlying mechanisms of its role in stemness, including drug resistance, remain to be fully elucidated. As a key regulator of mTORC1 activity, DDIT4 takes an essential role in the activation of cell autophagy in response to hypoxia or nutrient deprivation [32, 41]. It has been verified that autophagy participates in leukemia development and treatment resistance, and inhibition of autophagy can impair the stemness of leukemia stem cells [42, 43]. Here, we demonstrate that the HOXA cluster mediated the role of DDIT4 in self‐renewal and leukemogenesis, providing a new mechanism of DDIT4 regulated LSC stemness function. How DDIT4 regulates *HOXA* cluster gene expression and whether autophagy participates in this regulation remain to be determined.

In the analysis of *HOXA* upregulation induced by DDIT4 overexpression, different patterns of *HOXA* expression were observed between KG‐1a and Kasumi‐1 cells. This cell line‐specific response may be due to the different properties, including genetic backgrounds, of these cell lines. *HOXA* gene expression exhibits a strong correlation with the differentiation stage of hematopoietic cells [44]. In AML, elevated *HOXA* transcript levels have been reported in specific FAB subtypes [45]. Although both KG‐1a and Kasumi‐1 cells are CD34‐positive and originate from primitive differentiation stages, they are derived from different FAB subtypes, with KG‐1a representing a more differentiation‐resistant AML subtype compared with Kasumi‐1 [46]. *HOXA* genes are preferentially induced in AML derived from primitive hematopoietic cells, which may explain why DDIT4 upregulates a greater number of *HOXA* genes in KG‐1a cells.

Accumulating evidence has demonstrated that elevated *HOXA* gene expression is associated with poor prognosis in AML, and *HOXA* overexpression correlates with adverse clinical outcomes in AML patients [45, 47]. Although AML patients with t(8;21) are generally classified as a favorable‐risk subtype, a subset of these patients exhibits an increased risk of relapse [48, 49]. Cellular heterogeneity has been demonstrated among patients with t(8;21) AML [48]. Our findings reveal that DDIT4 upregulates *HOXA* expression in AML1‐ETO‐positive leukemia cells, suggesting that DDIT4‐high cells may represent a distinct chemoresistant subpopulation within AML1‐ETO leukemia.

## 5 Conclusion

In summary, our findings demonstrate that DDIT4 plays a critical role in leukemia development, regulates leukemia cell self‐renewal, and contributes to chemoresistance. Mechanistically, HOXA cluster specifically mediate the leukemia‐promoting effects of DDIT4. These observations suggest that DDIT4 may represent a potential therapeutic target for targeting leukemia stem cells in AML treatment.

## Conflict of interest

The authors declare no conflict of interest.

## Author contributions

YL performed all the experimental validation, analysis, and interpretation of data, and wrote the manuscript. ZC and HX helped with the establishment of a mouse AML transplantable model. ZX and YM analyzed the RNA‐seq data of the mouse. WL, JC, and HW analyzed the transcriptional signature of human AML patients. RG and SQ interpreted the data. MW supervised the study, interpreted data, and revised the manuscript. QR and JW designed and supervised the study, interpreted data, revised, and approved the manuscript.

## Supporting information


**Fig. S1.** Expression of *Ddit4* in AE9a leukemia cells co‐cultured with mouse MSC and MSC cell line.
**Fig. S2.** GSEA plots showing enrichment of genes in adult AML patients with high *DDIT4* expression (HOVON AML cohort).
**Fig. S3.** Effects of *DDIT4* expression in chemoresistance, G0 cell cycle phase and colony formation ability in AE9a leukemia cells.
**Fig. S4.** Immunophenotypes of the mice transplanted with AE9a‐transfected HSPCs‐*Ddit4* ^+/+^ or HSPCs‐*Ddit4* ^−/−^.
**Fig. S5.** Spontaneous apoptosis analysis in Kasumi‐1 and KG‐1a cells under DDIT4 overexpression.
**Fig. S6.** G0 phase of the cell cycle analysis in AE9a/*Ddit4* ^+/+^ and AE9a/*Ddit4* ^−/−^ cells co‐cultured with MC3T3‐E1 cells under Transwell‐based co‐culture and direct contact.
**Fig. S7.** Protein levels of DDIT4 in AE9a leukemia cells co‐cultured with mouse osteoblast cells under normoxic and hypoxic culture conditions.
**Table S1.** Primer sequences for quantitative RT‐PCR.

## Data Availability

The raw sequence data reported in this paper have been deposited in the GEO database under project accession numbers: GSE272010 and GSE272011. The supporting data related to our findings throughout our study will be available from the corresponding author [wangjx@ihcams.ac.cn] upon reasonable request.
