# Supplementary material for: The critical role of DNA damage‐inducible transcript 4 (DDIT4) in stemness character of leukemia cells and leukemia initiation
Source: Mol Oncol. 2025 Jul 7;19(11):3156–74. doi: 10.1002/1878-0261.70090 (PMC12591321; doi:10.1002/1878-0261.70090)
Supplement: Supplementary file 1 — Fig. S1. Expression of Ddit4 in AE9a leukemia cells co‐cultured with mouse MSC and MSC cell line. Fig. S2. GSEA plots showing enrichment of genes in adult AML patients with high DDIT4 expression (HOVON AML cohort). Fig. S3. Effects of DDIT4 expression in chemoresistance, G0 cell cycle phase and colony formation ability in AE9a leukemia cells. Fig. S4. Immunophenotypes of the mice transplanted with AE9a‐transfected HSPCs‐Ddit4 +/+ or HSPCs‐Ddit4 −/−. Fig. S5. Spontaneous apoptosis analysis in Kasumi‐1 and KG‐1a cells under DDIT4 overexpression. Fig. S6. G0 phase of the cell cycle analysis in AE9a/Ddit4 +/+ and AE9a/Ddit4 −/− cells co‐cultured with MC3T3‐E1 cells under Transwell‐based co‐culture and direct contact. Fig. S7. Protein levels of DDIT4 in AE9a leukemia cells co‐cultured with mouse osteoblast cells under normoxic and hypoxic culture conditions. Table S1. Primer sequences for quantitative RT‐PCR. [file MOL2-19-3156-s001.zip › mol270090-sup-0001-supinfo.docx]

**Supporting information**

**Supplementary Figure S1.** Expression of *Ddit4* in AE9a leukemia cells co-cultured with mouse MSC and MSC cell line. A. qRT-PCR analysis of relative expression of *Ddit4* in AE9a leukemia cells co-cultured with mouse MSC and OP9 cell. The data are presented as the mean±SEM from three independent experiments. The statistical significance were determined using Student unpaired *t*-test. ns: not significant. B. Representative Western blot image of protein levels of DDIT4 in AE9a leukemia cells co-cultured with mouse MSC and OP9 cell.

**Supplementary Figure S2.** GSEA plots showing enrichment of genes in adult AML patients with high *DDIT4* expression (HOVON AML cohort). The patients were divided into two groups based on the median expression level of *DDIT4.* High group: n =304, low group: n =304.

**Supplementary Figure S3.** Effects of DDIT4 expression in chemo-resistance, G0 cell cycle phase and colony formation ability in AE9a leukemia cells. A. Expression of *Ddit4* in AE9a cells in CBM from Ara-C treated or untreated mice. Expression level of *Ddit4* was determined by qRT-PCR and the data are presented as the mean±SEM from three independent experiments. ns: not significant. B. G0 phase of the cell cycle analysis on AE9a transduced HSPCs from WT and *Ddit4* knock out mice. G0 phase was analyzed in AE9a/*Ddit4*^+/+^ cells and AE9a/*Ddit4*^-/-^ cells cultured without MC3T3-E1. Left panel: Representative FACS plot for G0 phase analysis. Right panel: The percentage of cells in G0 phase. Data are presented as the mean±SEM from three independent experiments. ns: not significant. C. Colony formation of AE9a/*Ddit4*^+/+^ cells and AE9a/*Ddit4*^-/-^ cells cultured without MC3T3-E1. Data are presented as the mean ±SEM in one representative culture (n=3) of three independent experiments (1st: not significant; 2nd: p<0.05; 3rd: not significant).The statistical significance were determined using Student unpaired *t*-test (A,B,C).

**Supplementary Figure S4.** Immunophenotypes of the mice transplanted with AE9a-transfected HSPCs-*Ddit4*^+/+^ or HSPCs-*Ddit4*^-/-^. Representative FACS analysis of lineage marker expression in BM cells of AE9a-*Ddit4*^+/+^ (A) and AE9a-*Ddit4*^-/-^ mice (B).

**Supplementary Figure S5.** Spontaneous apoptosis analysis in Kasumi-1 and KG-1a cells under DDIT4 overexpression. Left panel: Representative FACS plots for apoptosis analysis based on PI and Annexin V-APC labeling. Right panel: The percentage of Annexin V^+^ cells. The data are presented as the mean±SEM from three independent experiments. The statistical significance were determined using Student unpaired *t*-test. ns: not significant.

**Supplementary Figure S6.** G0 phase of the cell cycle analysis in AE9a/*Ddit4*^+/+^ and AE9a/*Ddit4*^-/-^ cells co-cultured with MC3T3-E1 cells under Transwell-based coculture and direct contact. AE9a/*Ddit4*^+/+^ cells and AE9a/*Ddit4*^-/-^ cells were cocultured with MC3T3-E1 cells in Transwell inserts preventing cell contact or direct contact. A: Representative FACS plot for G0 phase analysis. B: The percentage of cells in G0 phase. Data are presented as the mean±SEM from three independent experiments. Each dot represents the value of the ratio of cells in G0 phase from one experiment. The statistical significance were determined using unpaired *t*-test. Exact p values for all pairwise comparisons are annotated above their corresponding bars. ns: not significant. Culture alone: AE9a cells without co-culture. Transwell: AE9a cells co-cultured with MC3T3-E1 osteoblasts using Transwell system (0.4 μm pore size). Direct contact: AE9a cells in direct physical contact with MC3T3-E1 osteoblasts.

**Supplementary Figure S7.** Protein levels of DDIT4 in AE9a leukemia cells co-cultured with mouse osteoblast cells under normoxic and hypoxic culture conditions. AE9a cells were incubated for 24h under hypoxia alone, osteoblast co-culture alone and hypoxia combined with osteoblast co-culture. Hypoxic culture condition was achieved by hypoxia-mimicking agent cobalt chloride (CoCl_2_) treatment (100μM). A. Representative Western blot image of protein levels of DDIT4 in AE9a leukemia cells under hypoxia alone, osteoblast co-culture alone and hypoxia combined with osteoblast co-culture. B. Relative protein levels of DDIT4. The values were calculated by the ratio of the band density of DDIT4 to that of internal control, and the band density was analyzed using Image J software. Data are presented as the mean±SEM from three independent experiments. The statistical significance were determined using Student unpaired *t*-test.

**Supplementary Table 1.** Primer sequences for quantitative RT-PCR
