# Supplementary material for: The critical role of DNA damage‐inducible transcript 4 (DDIT4) in stemness character of leukemia cells and leukemia initiation
Source: Mol Oncol. 2025 Jul 7;19(11):3156–74. doi: 10.1002/1878-0261.70090 (PMC12591321; doi:10.1002/1878-0261.70090)
Supplement: Supplementary file 1 — Fig. S1. Expression of Ddit4 in AE9a leukemia cells co‐cultured with mouse MSC and MSC cell line. Fig. S2. GSEA plots showing enrichment of genes in adult AML patients with high DDIT4 expression (HOVON AML cohort). Fig. S3. Effects of DDIT4 expression in chemoresistance, G0 cell cycle phase and colony formation ability in AE9a leukemia cells. Fig. S4. Immunophenotypes of the mice transplanted with AE9a‐transfected HSPCs‐Ddit4 +/+ or HSPCs‐Ddit4 −/−. Fig. S5. Spontaneous apoptosis analysis in Kasumi‐1 and KG‐1a cells under DDIT4 overexpression. Fig. S6. G0 phase of the cell cycle analysis in AE9a/Ddit4 +/+ and AE9a/Ddit4 −/− cells co‐cultured with MC3T3‐E1 cells under Transwell‐based co‐culture and direct contact. Fig. S7. Protein levels of DDIT4 in AE9a leukemia cells co‐cultured with mouse osteoblast cells under normoxic and hypoxic culture conditions. Table S1. Primer sequences for quantitative RT‐PCR. [file MOL2-19-3156-s001.zip › mol270090-sup-0009-TableS1.pdf]

Supplementary Table 1. Primer sequences for quantitative RT-PCR

Mouse

Gene	Forward (5' to 3')	Reverse (5' to 3')
DDIT4	CTTCGTCCTCGTCTCGAACT	CCATCCAGGTATGAGGAGTCTTCC
HOXA1	GAATACCCCATCCTTGGCAGTG	CCACTAGGAAGCGGTCGTCG
HOXA4	CTCTCGAACCGCCTATACCC	GATCGCATCTTGGTGTTGGG
HOXA5	CTCATTTTGCGGTCGCTATCC	TGAGATCCATGCCATTGTAGCC
HOXA6	ACACAAGCCCTGTTTACCCC	CTTTGGCCTCAGAGTCTTCCC
HOXA7	CACCCAACTCGCAGAGAAGC	TCCTGTCGGGTCCTGAACTG
HOXA9	CACCCCGACTTCAGTCCTTG	GCGGTTCAGGTTTAATGCCA
HOXA10	CCAACTGGCTCACAGCAAAGA	TCAGTTTCATCCTGCGATTCTG
GAPDH	TGCACCACCAACTGCTTAG	GGATGCAGGGATGATGTTC

Human

Gene	Forward (5' to 3')	Reverse (5' to 3')
DDIT4	GACACGGCTTACCTGGATGG	CGCAGTAGTTCTTTGCCCAC
HOXA1	CAGACTTCCGGGAACCTGG	GCTTCCTGATTTAACGCGTAGG
HOXA4	CACGCTCTGTTTGTCTGAGC	TGGAGGATCGCATCTTGGTG
HOXA5	GCGCAAGCTGCACATAAGTC	CGGAGAGGCAAAGAGCATGT
HOXA6	TCCACTTCAACCGCTACCTG	CTGGGCTGCGTGGAATTGA
HOXA7	CCACTTCAACCGCTACCTGA	CGGACCTTCGTCCTTATGCTC
HOXA9	TAAACCTGAACCGCTGTCGG	GCCTTCGCTGGGTTGTTTTT
HOXA10	CAAATGCCCCAAAGTCTCGG	AGAAAGGCGGAAGTAGCCAG
GAPDH	TGCACCACCAACTGCTTAG	AGAGGCAGGGATGATGTTC
