# Supplementary figures and images for: The critical role of DNA damage‐inducible transcript 4 (DDIT4) in stemness character of leukemia cells and leukemia initiation
Source: Mol Oncol. 2025 Jul 7;19(11):3156–74. doi: 10.1002/1878-0261.70090 (PMC12591321; doi:10.1002/1878-0261.70090)

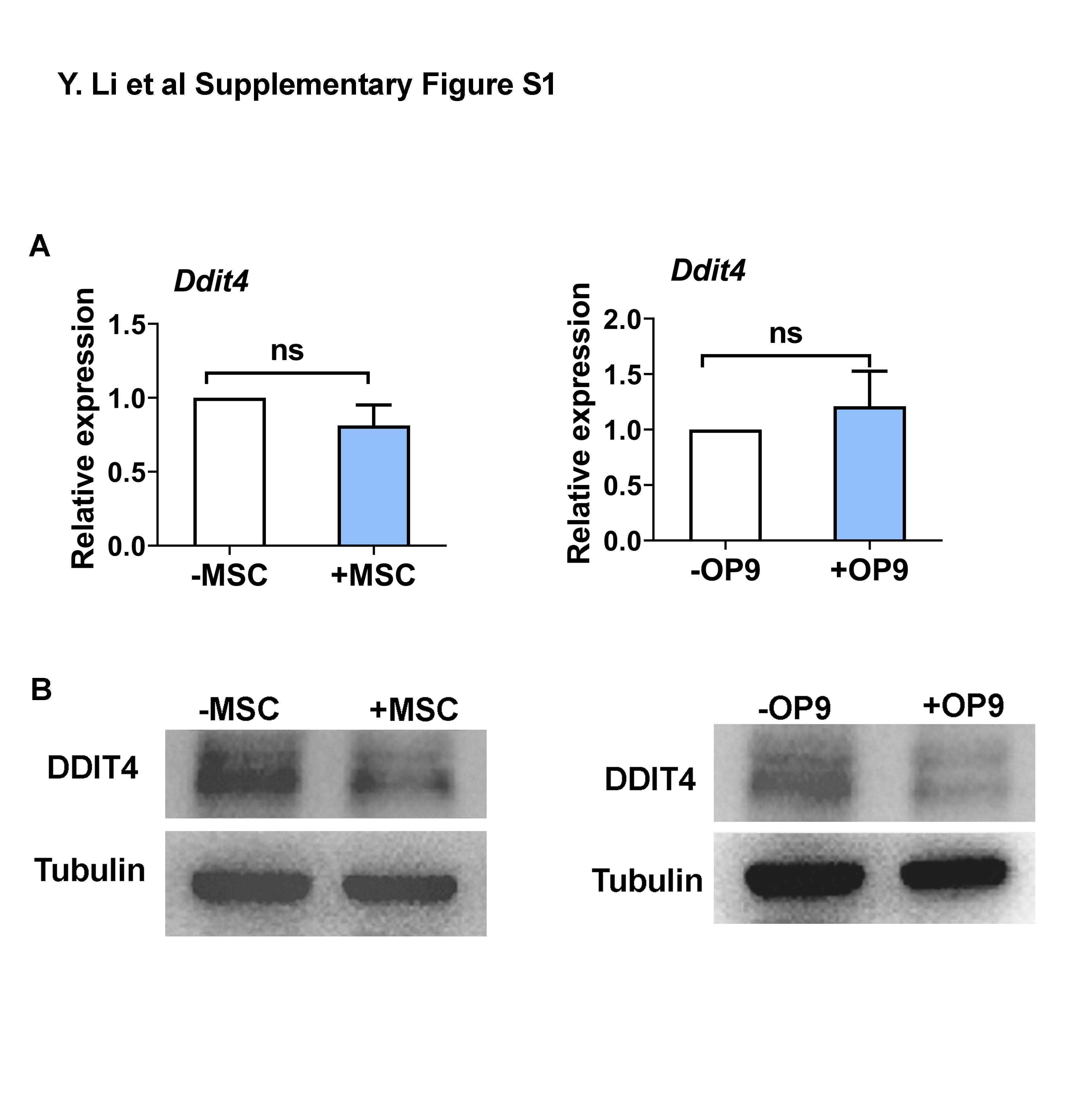

Supplement: Supplementary file 1 — Fig. S1. Expression of Ddit4 in AE9a leukemia cells co‐cultured with mouse MSC and MSC cell line. Fig. S2. GSEA plots showing enrichment of genes in adult AML patients with high DDIT4 expression (HOVON AML cohort). Fig. S3. Effects of DDIT4 expression in chemoresistance, G0 cell cycle phase and colony formation ability in AE9a leukemia cells. Fig. S4. Immunophenotypes of the mice transplanted with AE9a‐transfected HSPCs‐Ddit4 +/+ or HSPCs‐Ddit4 −/−. Fig. S5. Spontaneous apoptosis analysis in Kasumi‐1 and KG‐1a cells under DDIT4 overexpression. Fig. S6. G0 phase of the cell cycle analysis in AE9a/Ddit4 +/+ and AE9a/Ddit4 −/− cells co‐cultured with MC3T3‐E1 cells under Transwell‐based co‐culture and direct contact. Fig. S7. Protein levels of DDIT4 in AE9a leukemia cells co‐cultured with mouse osteoblast cells under normoxic and hypoxic culture conditions. Table S1. Primer sequences for quantitative RT‐PCR. [file MOL2-19-3156-s001.zip › mol270090-sup-0002-FigureS1.jpg]

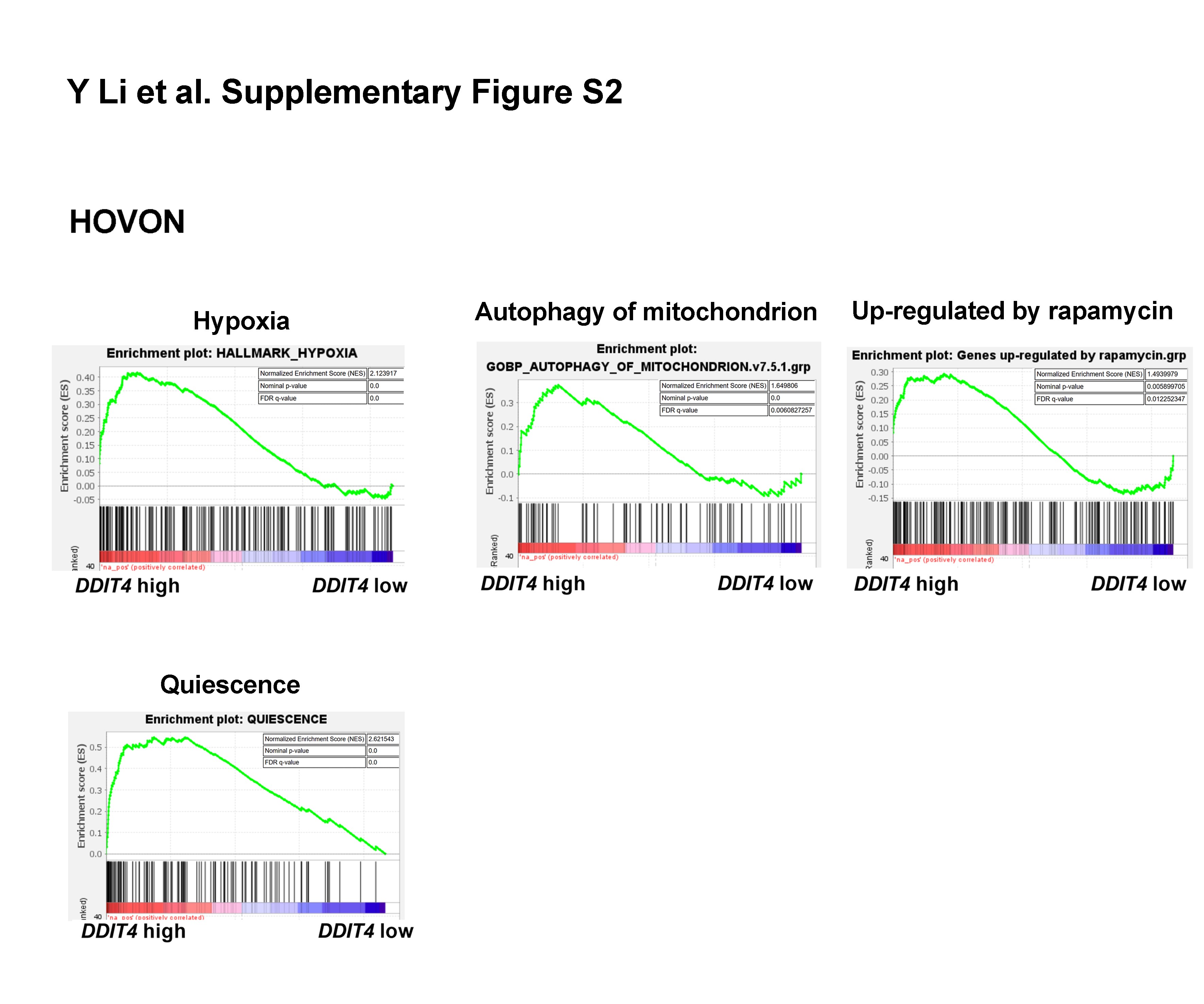

Supplement: Supplementary file 1 — Fig. S1. Expression of Ddit4 in AE9a leukemia cells co‐cultured with mouse MSC and MSC cell line. Fig. S2. GSEA plots showing enrichment of genes in adult AML patients with high DDIT4 expression (HOVON AML cohort). Fig. S3. Effects of DDIT4 expression in chemoresistance, G0 cell cycle phase and colony formation ability in AE9a leukemia cells. Fig. S4. Immunophenotypes of the mice transplanted with AE9a‐transfected HSPCs‐Ddit4 +/+ or HSPCs‐Ddit4 −/−. Fig. S5. Spontaneous apoptosis analysis in Kasumi‐1 and KG‐1a cells under DDIT4 overexpression. Fig. S6. G0 phase of the cell cycle analysis in AE9a/Ddit4 +/+ and AE9a/Ddit4 −/− cells co‐cultured with MC3T3‐E1 cells under Transwell‐based co‐culture and direct contact. Fig. S7. Protein levels of DDIT4 in AE9a leukemia cells co‐cultured with mouse osteoblast cells under normoxic and hypoxic culture conditions. Table S1. Primer sequences for quantitative RT‐PCR. [file MOL2-19-3156-s001.zip › mol270090-sup-0003-FigureS2.jpg]

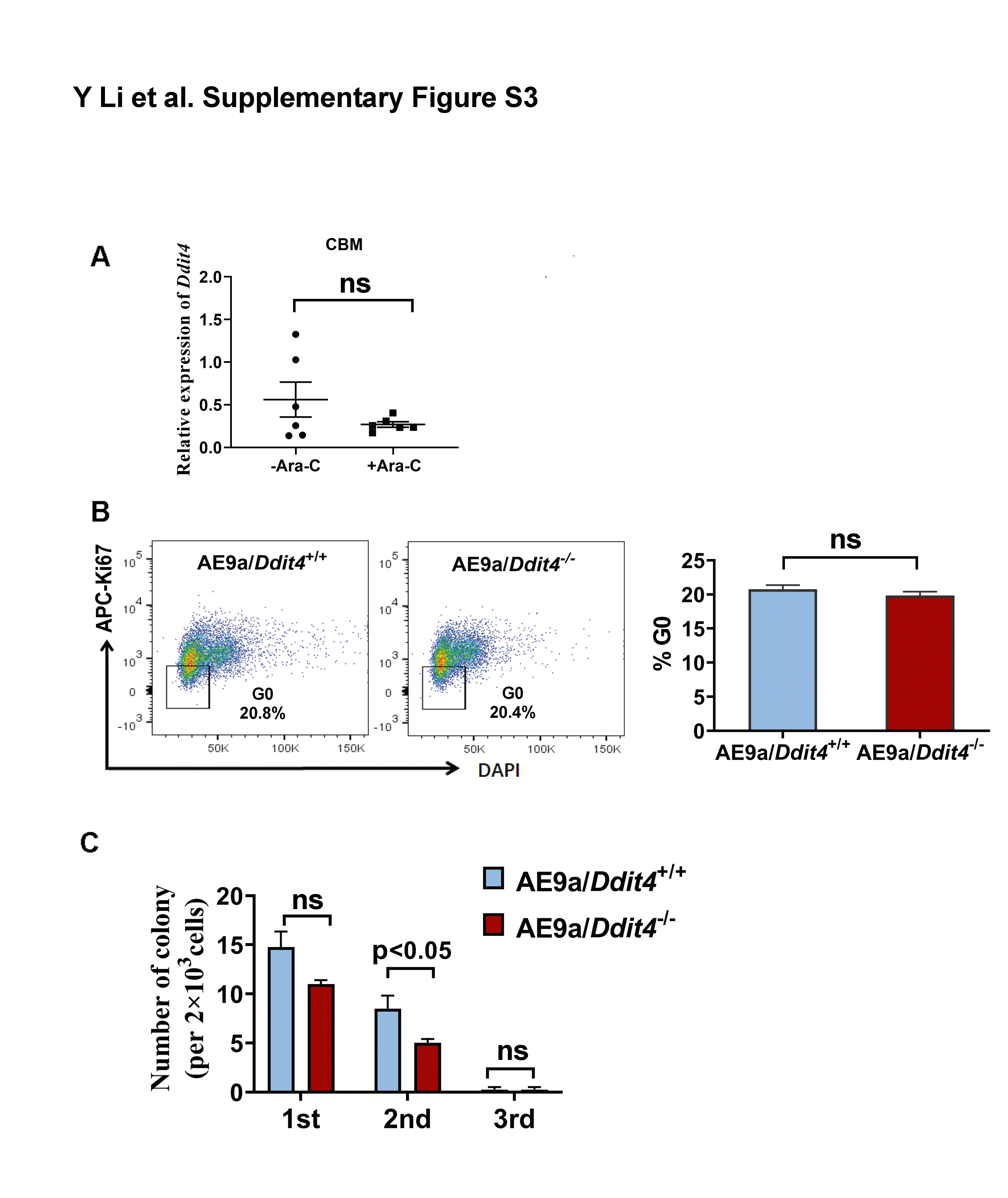

Supplement: Supplementary file 1 — Fig. S1. Expression of Ddit4 in AE9a leukemia cells co‐cultured with mouse MSC and MSC cell line. Fig. S2. GSEA plots showing enrichment of genes in adult AML patients with high DDIT4 expression (HOVON AML cohort). Fig. S3. Effects of DDIT4 expression in chemoresistance, G0 cell cycle phase and colony formation ability in AE9a leukemia cells. Fig. S4. Immunophenotypes of the mice transplanted with AE9a‐transfected HSPCs‐Ddit4 +/+ or HSPCs‐Ddit4 −/−. Fig. S5. Spontaneous apoptosis analysis in Kasumi‐1 and KG‐1a cells under DDIT4 overexpression. Fig. S6. G0 phase of the cell cycle analysis in AE9a/Ddit4 +/+ and AE9a/Ddit4 −/− cells co‐cultured with MC3T3‐E1 cells under Transwell‐based co‐culture and direct contact. Fig. S7. Protein levels of DDIT4 in AE9a leukemia cells co‐cultured with mouse osteoblast cells under normoxic and hypoxic culture conditions. Table S1. Primer sequences for quantitative RT‐PCR. [file MOL2-19-3156-s001.zip › mol270090-sup-0004-FigureS3.jpg]

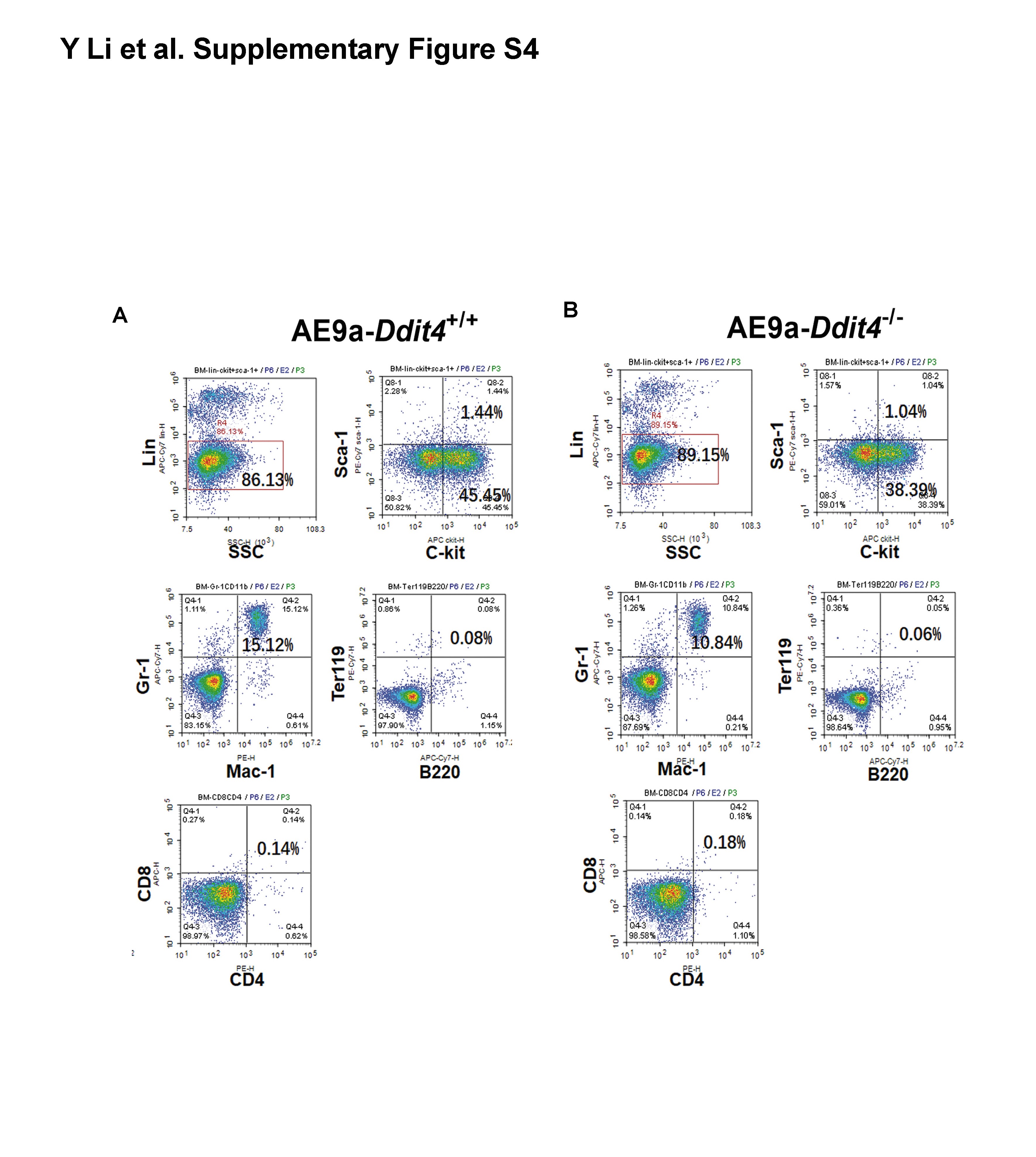

Supplement: Supplementary file 1 — Fig. S1. Expression of Ddit4 in AE9a leukemia cells co‐cultured with mouse MSC and MSC cell line. Fig. S2. GSEA plots showing enrichment of genes in adult AML patients with high DDIT4 expression (HOVON AML cohort). Fig. S3. Effects of DDIT4 expression in chemoresistance, G0 cell cycle phase and colony formation ability in AE9a leukemia cells. Fig. S4. Immunophenotypes of the mice transplanted with AE9a‐transfected HSPCs‐Ddit4 +/+ or HSPCs‐Ddit4 −/−. Fig. S5. Spontaneous apoptosis analysis in Kasumi‐1 and KG‐1a cells under DDIT4 overexpression. Fig. S6. G0 phase of the cell cycle analysis in AE9a/Ddit4 +/+ and AE9a/Ddit4 −/− cells co‐cultured with MC3T3‐E1 cells under Transwell‐based co‐culture and direct contact. Fig. S7. Protein levels of DDIT4 in AE9a leukemia cells co‐cultured with mouse osteoblast cells under normoxic and hypoxic culture conditions. Table S1. Primer sequences for quantitative RT‐PCR. [file MOL2-19-3156-s001.zip › mol270090-sup-0005-FigureS4.jpg]

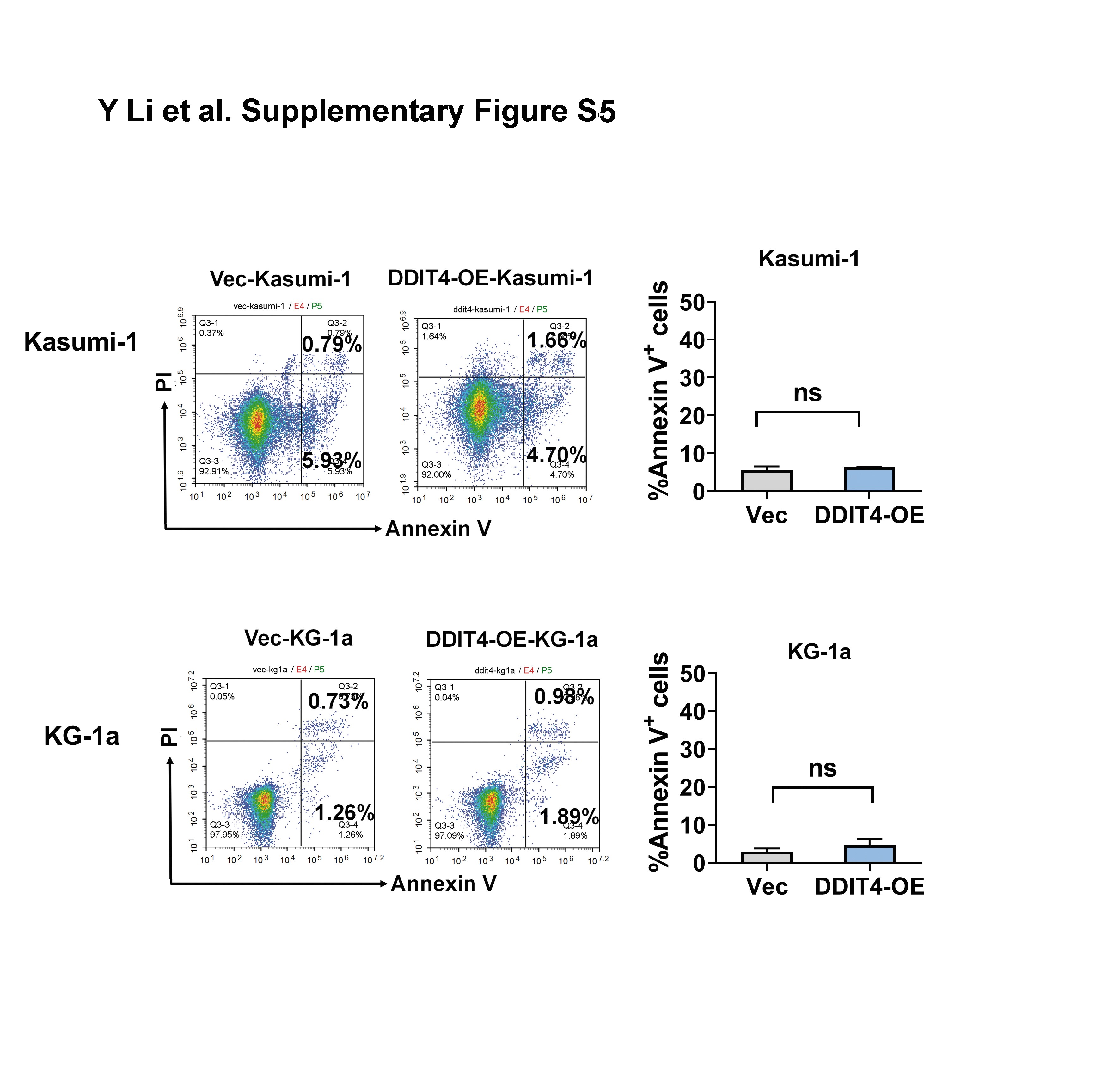

Supplement: Supplementary file 1 — Fig. S1. Expression of Ddit4 in AE9a leukemia cells co‐cultured with mouse MSC and MSC cell line. Fig. S2. GSEA plots showing enrichment of genes in adult AML patients with high DDIT4 expression (HOVON AML cohort). Fig. S3. Effects of DDIT4 expression in chemoresistance, G0 cell cycle phase and colony formation ability in AE9a leukemia cells. Fig. S4. Immunophenotypes of the mice transplanted with AE9a‐transfected HSPCs‐Ddit4 +/+ or HSPCs‐Ddit4 −/−. Fig. S5. Spontaneous apoptosis analysis in Kasumi‐1 and KG‐1a cells under DDIT4 overexpression. Fig. S6. G0 phase of the cell cycle analysis in AE9a/Ddit4 +/+ and AE9a/Ddit4 −/− cells co‐cultured with MC3T3‐E1 cells under Transwell‐based co‐culture and direct contact. Fig. S7. Protein levels of DDIT4 in AE9a leukemia cells co‐cultured with mouse osteoblast cells under normoxic and hypoxic culture conditions. Table S1. Primer sequences for quantitative RT‐PCR. [file MOL2-19-3156-s001.zip › mol270090-sup-0006-FigureS5.jpg]

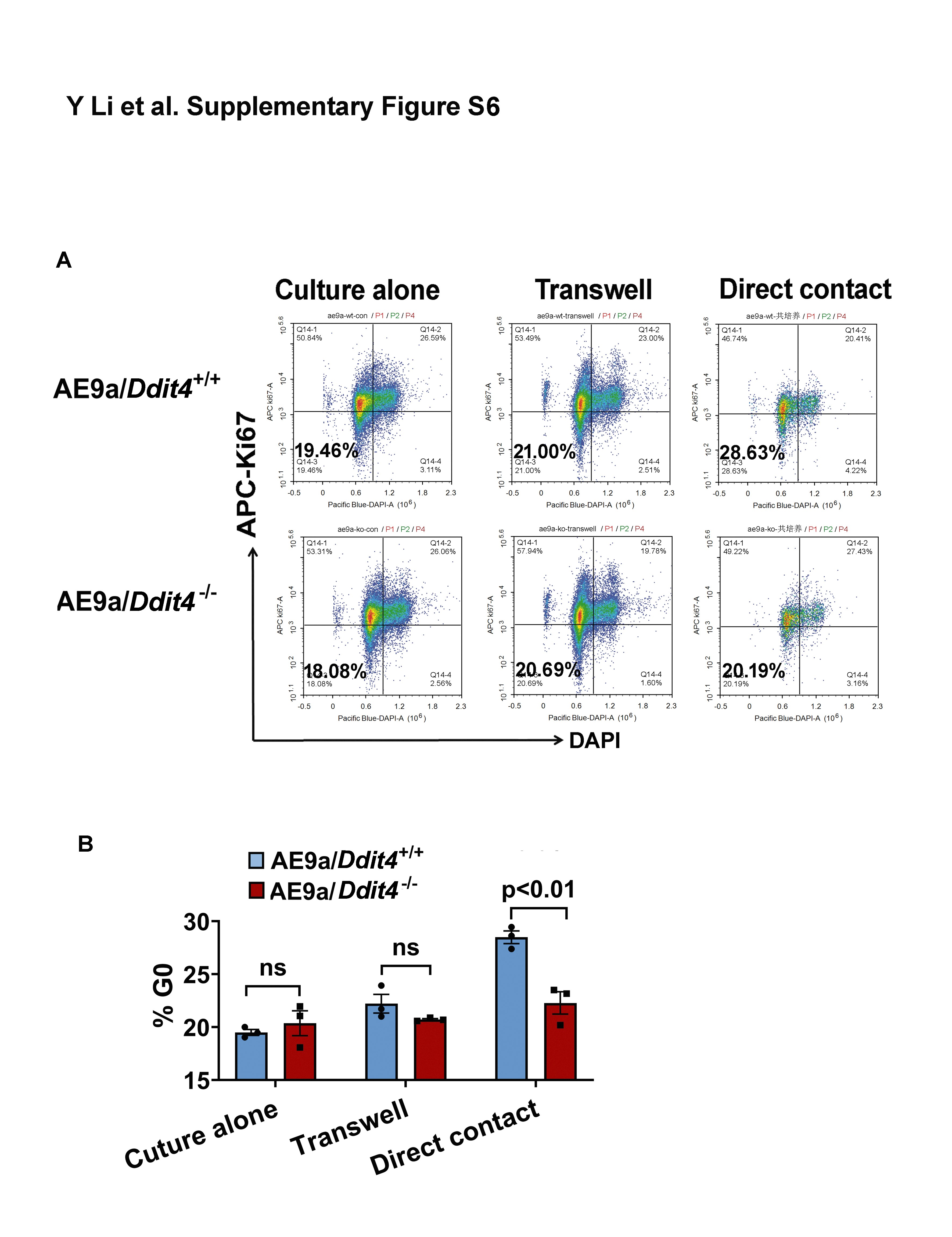

Supplement: Supplementary file 1 — Fig. S1. Expression of Ddit4 in AE9a leukemia cells co‐cultured with mouse MSC and MSC cell line. Fig. S2. GSEA plots showing enrichment of genes in adult AML patients with high DDIT4 expression (HOVON AML cohort). Fig. S3. Effects of DDIT4 expression in chemoresistance, G0 cell cycle phase and colony formation ability in AE9a leukemia cells. Fig. S4. Immunophenotypes of the mice transplanted with AE9a‐transfected HSPCs‐Ddit4 +/+ or HSPCs‐Ddit4 −/−. Fig. S5. Spontaneous apoptosis analysis in Kasumi‐1 and KG‐1a cells under DDIT4 overexpression. Fig. S6. G0 phase of the cell cycle analysis in AE9a/Ddit4 +/+ and AE9a/Ddit4 −/− cells co‐cultured with MC3T3‐E1 cells under Transwell‐based co‐culture and direct contact. Fig. S7. Protein levels of DDIT4 in AE9a leukemia cells co‐cultured with mouse osteoblast cells under normoxic and hypoxic culture conditions. Table S1. Primer sequences for quantitative RT‐PCR. [file MOL2-19-3156-s001.zip › mol270090-sup-0007-FigureS6.jpg]

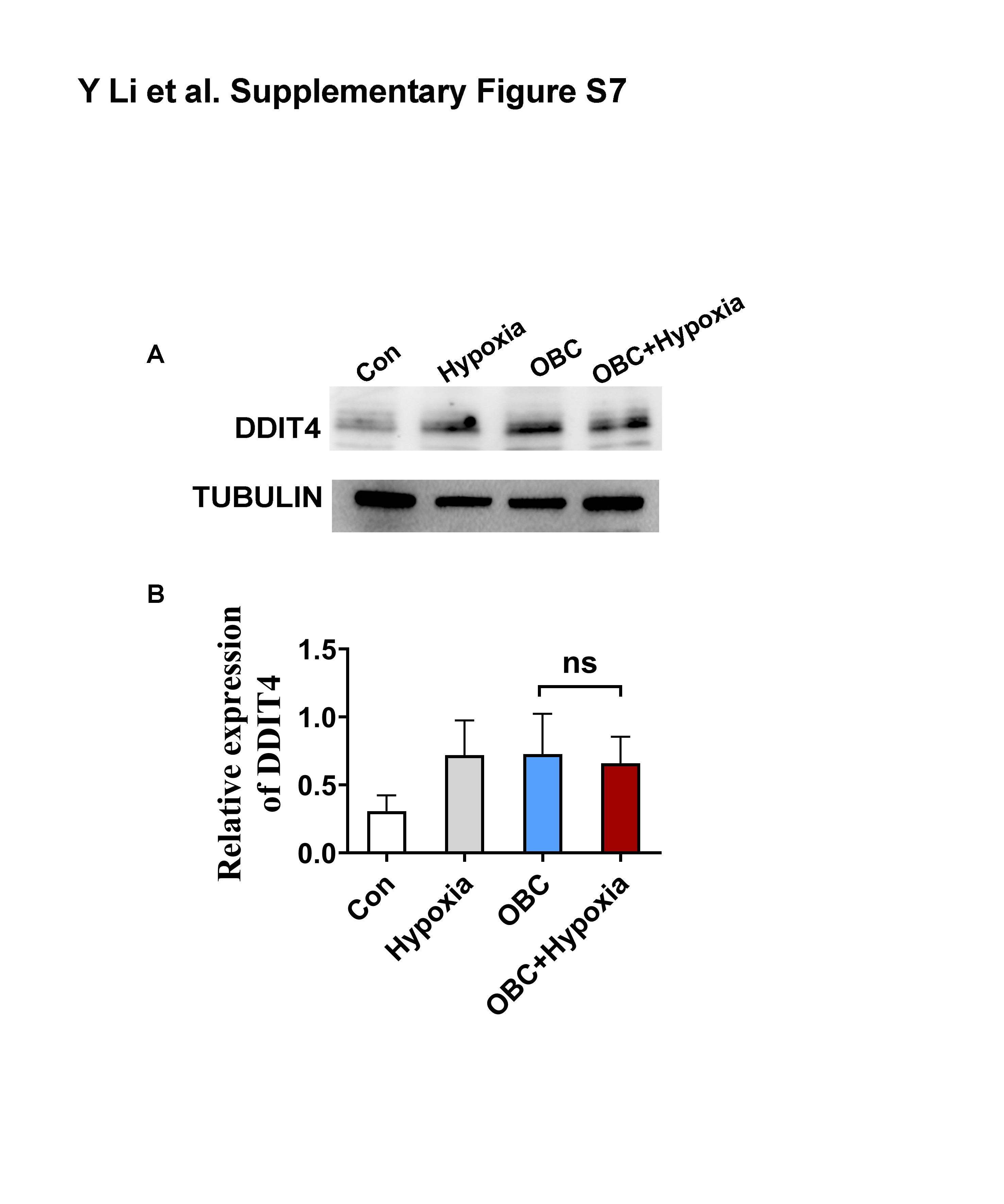

Supplement: Supplementary file 1 — Fig. S1. Expression of Ddit4 in AE9a leukemia cells co‐cultured with mouse MSC and MSC cell line. Fig. S2. GSEA plots showing enrichment of genes in adult AML patients with high DDIT4 expression (HOVON AML cohort). Fig. S3. Effects of DDIT4 expression in chemoresistance, G0 cell cycle phase and colony formation ability in AE9a leukemia cells. Fig. S4. Immunophenotypes of the mice transplanted with AE9a‐transfected HSPCs‐Ddit4 +/+ or HSPCs‐Ddit4 −/−. Fig. S5. Spontaneous apoptosis analysis in Kasumi‐1 and KG‐1a cells under DDIT4 overexpression. Fig. S6. G0 phase of the cell cycle analysis in AE9a/Ddit4 +/+ and AE9a/Ddit4 −/− cells co‐cultured with MC3T3‐E1 cells under Transwell‐based co‐culture and direct contact. Fig. S7. Protein levels of DDIT4 in AE9a leukemia cells co‐cultured with mouse osteoblast cells under normoxic and hypoxic culture conditions. Table S1. Primer sequences for quantitative RT‐PCR. [file MOL2-19-3156-s001.zip › mol270090-sup-0008-FigureS7.jpg]
